# Characterization of Phenolic Profiles Using UPLC-Q-TOF-MS/MS and NMR in the Biofunctional Fraction of Korean Winter Spinach (*Spinacia oleracea* L.) Leaves: Evaluation of Major Phenolics and Their Bioactivities Under Optimized Extraction Conditions

**DOI:** 10.3390/antiox15060686

**Published:** 2026-05-29

**Authors:** Eun Young Seo, Eun Jeong Ko, Du Yong Cho, Ye Ri Jeong, Se Hyeon Jeon, Dong Hyun Park, Mu Yeun Jang, Jeong Yoon Kim, Kye Man Cho, Jin Hwan Lee

**Affiliations:** 1 Department of Smart Green Resources, Dong-A University, 37, Nakdong-daero 550 beon-gil, Saha-gu, Busan 49315, Republic of Koreaejko9562@naver.com (E.J.K.); qurdb0107@naver.com (Y.R.J.); bbk6567@naver.com (S.H.J.); pdh000926@naver.com (D.H.P.); 2 Department of GreenBio Science and Agri-Food Bio Convergence Institute, Gyeongsang National University, Jinju 52727, Republic of Korea; dycho@gnu.ac.kr (D.Y.C.); jmy4330@naver.com (M.Y.J.); 3 Department of Pharmaceutical Engineering, Institute of Agricultural and Life Science (IALS), Anti-Aging Bio Cell Factory Regional Leading Research Center (ABC-RLRC), Gyeongsang National University, Jinju 52828, Republic of Korea; jykim21@gnu.ac.kr

**Keywords:** spinach leaves, phenolic, biofunctional activity, optimal extraction condition, UPLC-Q-TOF-MS/MS, HPLC, NMR

## Abstract

This study is the first to demonstrate fluctuations in major phenolics and biofunctional properties under various extraction conditions of Korean winter spinach (*Allseason cultivar*) leaves. In contrast to earlier reports on summer- or greenhouse-cultivated spinach, which mainly relied on HPLC-DAD or LC-MS profiling and one or two bioactivity assays, the present work combines UPLC-Q-TOF-MS/MS with NMR-based structural confirmation of three major flavone glucuronides (**11**–**13**) and integrates five complementary bioactivity assays (DPPH, ABTS, FRAP, DNA protection, and tyrosinase inhibition) within a single optimization framework. A 50% methanol extract yielded twelve phenolics (patuletin, spinacetin, spinatoside, jaceidin, and methylenedioxyflavone-glucuronide derivatives) elucidated by UPLC-Q-TOF-MS/MS, with the isolated major phenolics **11**–**13** further verified by NMR. Total phenols and total flavonoids of biofunctional characteristics varied significantly depending on the solvent system. The optimal extraction conditions (50% methanol, 72 h, 35 °C) resulted in the highest phenolic levels of phenolics **11**–**13** (total: ~6.5 mg/g) and bioactivities (DNA protection > ABTS > tyrosinase inhibition > FRAP > DPPH, at 500 μg/mL). PCA and hierarchical clustering distinguished extraction profiles, with 50–70% methanol extracts forming clear clusters. Among the isolated phenolics, phenolic **12** showed the strongest antioxidant activity (DPPH IC_50_ = 57.6 μM; ABTS IC_50_ = 21.9 μM). These findings suggest that spinach leaves are a valuable source of bioactive phenolics for nutraceutical applications under optimized extraction conditions.

## 1. Introduction

Phenolic phytochemicals, a class of aromatic secondary metabolites, are widely distributed in natural plant sources, including crops, fruits, vegetables, herbs, and other edible materials [1,2,3,4,5,6,7]. These constituents have been extensively studied due to their broad range of health-promoting properties, such as antioxidant, antityrosinase, antidiabetic, anticancer, anti-inflammatory, and antiatherosclerotic effects [8,9,10,11,12,13]. Recent research has focused on diverse subclasses of phenolics, namely phenolic acids, flavonoids, xanthones, chalcones, and anthocyanins [1,5,6,14,15]. These bioactive, particularly those derived from phenolic-rich plants, play critical roles in regulating cellular oxidative stress and in scavenging reactive oxygen species (ROS), thereby mitigating oxidative damage and the progression of chronic diseases [7,11,12,16,17]. In addition, ROS are implicated in skin-related conditions such as hyperpigmentation, primarily by promoting melanocyte proliferation and increasing melanin synthesis [16,18]. Phenolic antioxidants capable of scavenging ROS may also act as inhibitors of melanogenesis, providing dual benefits for both health and cosmetic applications [6,18]. Although numerous phenolic phytochemicals exhibit antioxidant capacities, their reducing abilities and bioefficacy can vary significantly, depending on molecular structures and extraction conditions. For these considerations, evaluating the antioxidant and enzyme inhibitory properties of phenolic-rich extracts is essential for understanding their biofunctional potential and supporting their application in health-related industries [7,8,9,19].

Recent studies have emphasized the simultaneous enhancement of multiple bioactivities through optimized extraction conditions [9,20,21,22,23]. Extraction is a commonly used technique to obtain active constituents from complex matrices [4,24]. Conventional techniques such as solvent extraction and hydrothermal processes have been widely applied to isolate phenolic phytochemicals [3,7,25,26]. However, these approaches often suffer from limitations, including long processing times and low selectivity [12,14,23]. Although modern extraction strategies such as ultrasound-, high-speed shearing-, and microwave-assisted extraction (UAE, HSE, and MAE) offer shorter processing times and improved efficiency [15,21,27], they often require costly instrumentation and may compromise thermally unstable compounds. Optimizing conventional extraction parameters (solvent composition, time, temperature, and pH) provides a more flexible and scalable approach [7,9,14,20,25]. By controlling these variables, researchers can enhance extraction yields and preserve the structural integrity of heat-sensitive phenolics without complex equipment. Numerous studies have emphasized the significance of optimizing conditions to maximize the recovery of bioactive compounds for food, cosmetic, and pharmaceutical applications.

Among various edible plants, spinach (*Spinacia oleracea* L.), a member of the Chenopodiaceae family, is widely cultivated and consumed worldwide [2,8,12,25,28]. This crop is economically important and is a nutritious green leafy vegetable enriched with essential vitamins, dietary fibers, minerals, and phytochemicals, which can be used fresh or processed in the human diet [8,25,26,29]. In particular, while common spinach is recognized for its beneficial phenolic content, winter spinach is noted for its enhanced nutritional value and unique properties, highlighting the need for further research to explore its potential benefits. Due to its significant nutritional profile, spinach is frequently recommended as a supplementary food to promote human health [30]. Additionally, spinach is well-known for being a rich source of phenolic phytochemicals and exhibits notable functional properties, including antiproliferative, anti-inflammatory, antioxidant, anti-obesity, and lipid-lowering activities [8,30,31]. Despite the established benefits of spinach, limited information is available regarding the comparative evaluation of phenolic phytochemicals based on their bioactive properties. Therefore, we examined the beneficial characteristics of suitable phenolic metabolites and their functional attributes for applications in the food and nutraceutical industries, specifically focusing on winter spinach leaves. In this study, our evaluation was extended beyond fundamental antioxidant assays to encompass DNA protective and tyrosinase inhibitory activities. These assays were strategically integrated to assess the multifunctional potential of winter spinach for nutraceutical and cosmeceutical applications, focusing on the preservation of genomic integrity and the modulation of enzymatic pathways such as melanogenesis.

The primary objectives of the current work were to identify high-value phenolic materials with strong biofunctional activities under optimized extraction conditions. Phenolic profiling was performed by UPLC-Q-TOF-MS/MS, the structures of the three major flavone glucuronides were confirmed by NMR spectroscopy, and five complementary biofunctional assays (DPPH, ABTS, FRAP, DNA protection, and tyrosinase inhibition) were integrated under systematically optimized extraction conditions (solvent, temperature, time, and pH). To our knowledge, this is the first study to combine these three analytical and biofunctional dimensions for Korean winter spinach leaves.

## 2. Materials and Methods

### 2.1. Plant Material and Chemicals

For this study, the most commonly cultivated Korean spinach, Allseason (known as Sagyejul in Korean), was chosen. This source is a commercially improved variety designed for year-round cultivation in the Republic of Korea. The seeds were purchased from Asia Seed Co., Ltd. (Seoul, Republic of Korea) and were sown on 10 December 2022, in the experimental fields located at Gyeongsang National University, Jinju, Republic of Korea. The spinach leaves were harvested on 15 March 2023, and were immediately washed using sterile water to remove dust. To eliminate residual moisture and minimize the risk of oxidation while preserving the integrity of phytochemicals, the leaves were air-dried in a shaded environment at room temperature for 3 days. The dried leaves were then freeze-dried and stored at −70 °C until they were needed for analysis. Folin–Ciocalteu phenol reagent, sodium hydroxide, sodium nitrite, sodium carbonate, diethylene glycol, 2,2-diphenyl-1-picrylhydrazyl (DPPH), 2,2′-azino-bis(3-ethylbenzthiazoline-6-sulphonic acid) (ABTS), butylated hydroxytoluene (BHT), 6-hydroxy-2,5,7,8-tetramethylchroman-2-carboxylic acid (Trolox), 2,4,6-tripyridyl-*s*-triazine (TPTZ), tyrosinase (EC 1.14.18.1), ascorbic acid, gallic acid, quercetin, acetate buffer, potassium persulphate, Sephadex LH-20, and deuterated methanol (CD_3_OD) were purchased from Sigma Chemical Co. (St. Louis, MO, USA). The pUC18 super-coiled plasmid DNA was sourced from Thermo Fisher (Waltham, MA, USA). Solvents used in the experiments, including analytical-grade methanol, ethyl acetate, *n*-hexane, chloroform, ethanol, acetonitrile, and water, were obtained from J.T. Baker (Phillipsburg, NJ, USA). Silica gel (Kieselgel 60, 70–230 and 230–400 mesh) and F254 thin layer chromatography (TLC) glass plates were acquired from Merck (Darmstadt, Germany). HPLC grade water and methanol were also provided by J.T. Baker. All other analytical-grade reagents utilized in this research were obtained from Sigma-Aldrich (St. Louis, MO, USA).

### 2.2. Instruments

To evaluate the antioxidant and tyrosinase inhibitory effects, UV-Vis absorption spectra were obtained using an Agilent BioTeck microplate spectrophotometer (EPOCH2; BioTek Instruments, Winooski, VT, USA). The total phenolic content (TPC) and total flavonoid content (TFC) analyses were also measured using this spectrometer. Phenolic metabolite profiling was conducted using ultra-performance liquid chromatography (ACQUITY UPLC™, Waters, Milford, MA, USA), equipped with a photodiode array (PDA, Waters) coupled to a quadrupole time-of-flight mass spectrometer (Micromass QTof Premier™ Mass Spectrometer, Waters). The isolated phenolic metabolites were elucidated using a Bruker AM 500 spectrometer (Bruker, Karlsruhe, Germany). Quantitative analysis of phenolic phytochemicals (**11**, **12**, and **13**) was carried out with an HPLC Agilent 1100 system (Boeblingen, Germany), which consisted of an Agilent 1100 diode-array detector, a quaternary pump, and an autosampler.

### 2.3. Extraction and Isolation of Three Major Phenolic Derivatives

The isolations of three major phenolics were conducted using column chromatography and preparative HPLC analysis, as previously described in our data [5,10]. The dried spinach leaves (1.5 kg) were ground using a milling machine (J World Tech. Co., Ltd., Seoul, Republic of Korea) for 5 min. The pulverized leaves (1 kg) were extracted with MeOH in three extraction cycles, each using 1.5 L of solvent, in a shaking incubator for 7 days at 25 °C. The combined methanol extract was concentrated using a rotary evaporator at 40 °C to yield dark green gum. The resulting residue (190 g) was chromatographed on silica gel (8 cm × 60 cm, 230–400 mesh, 890 g) using CHCl_3_-acetone mixtures (*v*/*v*) [15:1 (1.2 L) → 10:1 (1.2 L) → 7:1 (1.2 L) → 5:1 (1.2 L) → 2:1 (1.2 L) → 1:1 (1.2 L)] and CHCl_3_-MeOH mixtures (*v*/*v*) [10:1 (1.2 L) → 7:1 (1.2 L) → 4:1 (1.2 L) → 2:1 (1.2 L) → 1:1 (1.2 L)] to generate 54 fractions (F1–F54), based on the comparison of phytochemical spots in TLC patterns using a UV lamp (254 nm; Spectronics Corporation, Westbury, NY, USA). Fractions F17–F20 (900 mg) were further fractionated by silica gel column chromatography (2.5 cm × 40 cm, 230–400 mesh, 30 g) with CHCl_3_-acetone (10:1 → 1:2, *v*/*v*) and CHCl_3_-MeOH (12:1 → 5:1, *v*/*v*) to yield 37 subfractions. Subfractions 25–30 (121 mg) were evaporated and re-chromatographed on silica gel (1.2 cm × 25 cm, 230–400 mesh, 20 g) with CHCl_3_-MeOH (10:1 → 6:1, *v*/*v*) to yield phenolic **13** (21 mg). Fractions F29–F33 (1.3 g) were subjected to column chromatography on a silica gel (2.5 cm × 45 cm, 230–400 mesh, 43 g) using a CHCl_3_-MeOH gradient system, starting from a 20:1 ratio and gradually increasing the polarity to 4:1 to produce 36 subfractions. Subfractions 17–22 (390 mg) were further purified by MPLC using a gradient elution of CH_3_CN in water (5 → 40%, 20 mL/min). The resulting eluate was subsequently fractionated using Sephadex LH-20 column chromatography with 70% methanol, yielding phenolic **12** (16 mg). In addition, subfractions 28–30 (260 mg) were chromatographed on a Sephadex LH 20 column (1.5 cm × 70 cm, 40 g) with 70% MeOH as the eluent, leading to the isolation of phenolic **11** (27 mg).

### 2.4. Evaluation of Total Phenolic and Flavonoid Contents

The materials for TPC and TFC (mg/100 g) were prepared as described in Section 2.3, using 1 g of dried sample extracted with 20 mL of solvents. TPC was quantified using the Folin–Ciocalteu reagent with minor modifications, as previously reported [5,19]. TFC was demonstrated by a colorimetric assay based on the method described by Mduda et al. [19]. For the TPC assay, 500 μL of the sample extract was mixed with 250 μL of 2 N Folin–Ciocalteu’s reagent and 250 μL of distilled water, followed by incubation at 25 °C for 1 h. Subsequently, 500 μL of 20% sodium carbonate solution was added, and the mixture was incubated for 10 min at 35 °C. The absorbance was measured at 750 nm using a UV-Vis spectrophotometer, and the phenolic content was calculated based on a gallic acid calibration curve (0.5–500 μg/mL). The results were expressed as milligrams of gallic acid equivalents (GAE) per 100 g of dried sample. The TFC method was conducted by adding 250 μL of the extract to 2 mL of methanol, followed by the addition of 1 mL of 90% diethylene glycol and 1 mL of 4 M NaOH. The mixture was incubated at 35 °C for 10 min, and absorbance was evaluated at 420 nm. TFC was quantified using a quercetin standard curve (0.5–500 μg/mL) and expressed as milligrams of quercetin equivalents (QE) per 100 g of dried sample. All measurements for TPC and TFC were performed in triplicate and repeated in three independent experiments. The results are reported as mean values.

### 2.5. Elucidation of Chemical Structures Using NMR Spectroscopy

Three isolated phenolic compositions were analyzed using nuclear magnetic resonance (NMR) spectroscopy with deuterated methanol (CD_3_OD) as the solvent and tetramethylsilane (TMS) as the internal standard in 5 mm NMR tubes, as previously described [5]. All chemical shifts in the NMR spectra were calibrated using the TMS signal as an internal reference. The NMR spectroscopic data, including ^1^H, ^13^C, distortionless enhancement by polarization transfer (DEPT) 90/135, ^1^H–^1^H correlated spectroscopy (COSY) and heteronuclear single quantum coherence (HSQC), were expressed in ppm and coupling constant (*J*) with Hertz [5]. Spectra were acquired using a Silicon Graphics O2 workstation running XWINNMR version 2.1 (Bruker, Rheinstetten, Germany). Chemical structures of phenolics **11**, **12**, and **13** were established from the interpretation of the NMR spectroscopic data, molecular ion values from mass spectrometry, and comparison with previously reported structures of similar metabolites [12,28,30,32,33].

### 2.6. Preparation of Samples and Calibration Curves for Quantification of Isolated Phenolics Using HPLC

The contents of the three main phenolics in spinach leaves were evaluated by HPLC analysis. Their calibration curves were generated using the methodologies described by Lee et al. [33]. Stock solutions of each standard (1 mg/mL) were prepared in methanol, and calibration was performed by plotting peak areas against eight different concentrations (0.5, 1, 5, 10, 25, 50, 100, and 250 μg/mL) measured at 340 nm, using Agilent ChemStation software (ChemStation Edition C.01.09; Agilent Technologies, Palo Alto, CA, USA). All calibration curves showed excellent linearity, with correlation coefficients (*r*^2^) exceeding 0.999. The standard calibration curves, regression equations, and coefficients of determination (*r*^2^) for the three isolated phenolics **11**–**13** were as follows: peak 11 (*y* = 12.08*x* + 6.70; *r*^2^ = 0.9999), peak 12 (*y* = 8.95*x* + 21.58; *r*^2^ = 0.9992), and peak 13 (peak 3, *y* = 12.14*x* + 29.28; *r*^2^ = 0.9991). Validation parameters for the HPLC quantification of phenolics **11**–**13** were assessed as follows: the Limit of Detection (LOD) was estimated to be 0.071 μg/mL; the Limit of Quantification (LOQ) was determined to be 0.245 μg/mL; precision was calculated with a relative standard deviation (RSD) of 2.3% based on three replicates; and recovery rates achieved were approximately 94% based on spiking experiments. For sample preparation, spinach leaves were finely ground for 3 min using an HR 2860 coffee grinder (Philips, Drachten, The Netherlands). Subsequently, 1.0 g of the pulverized sample was extracted with 20 mL of 50% methanol (or an alternative solvent) at 35 °C (or an alternative temperature) for 72 h (or adjusted durations). The resulting extract was centrifuged at 3000× *g* for 3 min to remove insoluble material and then passed through a 0.45 μm syringe filter (Whatman, Maidstone, UK) to obtain the clarified supernatant. The phenolic contents were determined using a reverse phase C18 column (ZORBAX Eclipse XDB C18, 250 mm × 4.6 mm, I.D., 5 μm, Agilent Technologies, Palo Alto, CA, USA) by an Agilent 1100 HPLC. Gradient elution was performed using solvent A (0.1% formic acid in water) and solvent B (acetonitrile) under the following conditions: 0–5 min, 10% B; 25 min, 20% B; 35 min, 55% B; 45 min, 65% B; and 55 min, 75% B, and was maintained for an additional 5 min before re-equilibrating to the initial conditions. Other chromatographic separation conditions were carried out at 25 °C column with a flow rate of 1 mL/min, and detection was monitored at 330 nm. The isolated phenolic phytochemical contents are measured as microgram per g of dried spinach leaves.

### 2.7. Conditions of UPLC-Q-TOF-MS/MS for Phenolic Profiles

The UPLC separation was performed on an ACQUITY BEH C18 chromatography column (100 mm × 2.1 mm, i.d., 1.7 μm, Waters, Milford, MA, USA). The mobile phase consisted of two solvents: (A) water containing 0.1% formic acid and (B) acetonitrile. The metabolites were separated using the following gradient elution profile: 0–1 min; 10% B; 1–3 min; 30% B; 3–4 min; 50% B; 4–5.5 min; 70% B; 5.5–7 min; 80% B; 7–8 min; 100% B. The flow rate was 0.35 mL/min, with an injection volume of 1 μL, and the column temperature was maintained at 25 °C. After chromatographic separation, the mobile phase was introduced directly into the Triple TOF X500R (SCIEX, Framingham, MA, USA) instrument coupled to the UPLC system. The MS system was operated in negative ion modes, with the mass range set at *m*/*z* 50–1500 *Da* at full scan resolution. The operating MS conditions were as follows: source temperature, 100 °C; capillary voltage, 3 kV; desolvation temperature, 400 °C; sample cone voltage, 30 V; cone gas flow, 30 L/h; desolvation gas flow, 800 L/h; collision energy, 10 eV; nebulizer gas 50 °C (GS1 50 and GS2 50). Data acquisition and control were performed using Analyst TF 1.7 software (AB SCIEX, Framingham, MA, USA) operating in information-dependent acquisition mode. A reference solution of leucine-enkephalin ([M–H]^−^, *m*/*z* 554.2615) was introduced via a sprayer and used as the lock mass. Metabolites were identified based on retention time, accurate mass, MS/MS fragmentation patterns, and elemental composition, supported by previous reports on spinach and database searches using SCIEX OS (v2.0; AB SCIEX, Foster City, CA, USA), ChemSpider (https://www.chemspider.com, accessed on 4 August 2024), MassBank (https://massbank.eu, accessed on 20 September 2024), and the Phytochemical Dictionary was used as a published reference.

### 2.8. Determination of Antioxidant and Tyrosinase Inhibition Properties

The radical scavenging capacities (DPPH and ABTS) and FRAP assays were carried out using the earlier-reported procedures with some modifications [10,17,19]. To compare extraction efficiencies of different solvents, powdered leaves (1.0 g, 60 mesh) were extracted with 10 mL of methanol for 12 h at room temperature. After filtration through Whatman No. 42 filter paper, the supernatant was collected for measurement. Based on the results obtained with different solvents, the extraction conditions of spinach leaves were optimized to 50% methanol at 35 °C for 72 h. Under these conditions, antioxidant and tyrosinase inhibitory activities were evaluated at various concentrations. To evaluate the antioxidant effect via the DPPH assay, a DPPH solution (1 mM) was prepared and adjusted to an absorbance of 0.70 at 517 nm. Sample extracts of spinach leaves (100 μL) or a positive control (BHT, 100 μL) at various concentrations were added to 3.9 mL of the DPPH solution. The reaction mixtures were incubated in the dark at 25 °C for 30 min. After incubation, absorbance was measured at 517 nm. The DPPH radical scavenging activity was documented as a percentage using the following equation:

Scavenging activity (%) = [(1 − As/Ac)] × 100(1)

where As is the absorbance of the sample and Ac is the absorbance of the control.

The ABTS radical scavenging activity was determined based on the ability of the samples to quench the ABTS^•+^ radical cation, using Trolox as the positive control. The ABTS^•+^ radical cation was generated by reacting a 7 mM ABTS stock solution with 2.45 mM potassium persulfate and allowing the mixture to stand in the dark for 10 h. The resulting solution was diluted with ethanol to achieve an absorbance of 0.70 at 734 nm. Subsequently, 0.9 mL of the ABTS^•+^ solution was mixed with 0.1 mL of each sample at different concentrations, and absorbance was measured using a spectrophotometer. The scavenging capacity was calculated as a percentage by the following equation:

Scavenging activity (%) = [(1 − As/Ac)] × 100(2)

where As is the absorbance of the sample and Ac is the absorbance of the control.

The FRAP reagent was formulated by mixing 2.5 mL of 10 mM TPTZ solution, 25 mL of 300 mM acetate buffer (pH 3.6), and 2.5 mL of 20 mM FeCl_3_, following the method described in previous studies [13,17,19]. The mixture was incubated for 15 min at 37 °C to allow reagent activation. Subsequently, 0.05 mL of the diluted sample was combined with 0.95 mL of the FRAP reagent and incubated again at 37 °C for 15 min. Absorbance was measured at 593 nm, and the antioxidant activity was compared with that of the positive control, ascorbic acid.

The inhibitory capacity of tyrosinase was evaluated based on previously reported methods [6,10], with minor modifications. For the tyrosinase inhibition assay, 30 μL of the diluted spinach sample or ascorbic acid (used as a positive control) was mixed with 970 μL of 0.05 mM sodium phosphate buffer (pH 6.8) in methanol. Subsequently, 1 mL of L-tyrosine solution was added, followed by 1 mL of tyrosinase (200 units/mL), and the mixture was vortexed for 1 min. The reaction was incubated for 30 min at 37 °C. Absorbance was then measured at 490 nm using a spectrophotometer. The tyrosinase inhibitory activity was determined as percentage ratios:

Inhibition (%) = [(Ac − As)/Ac] × 100(3)

where As: absorbance of sample and Ac: absorbance of control.

### 2.9. Measurement of DNA Protection Rate

To assess the DNA protective capacity of solvent extracts from spinach leaves, the metal-catalyzed oxidation (MCO) DNA cleavage assay was performed, following the methods reported by Yilmaz-Ozden et al. [13]. Supercoiled plasmid DNA (pUC18 from *E. coli*, 50 μg/mL) was diluted in phosphate-buffered saline (0.5 M, pH 7.4). The diverse concentrations of the extract (μg/mL; 5 μL each) were combined with plasmid DNA (5 μL), dithiothreitol (3.3 mM, 5 μL), and FeCl_3_ (15.4 μM, 5 μL), followed by incubation for 2 h at 37 °C. After incubation, 5 μL of the reaction mixture was mixed with 1 μL of DNA loading buffer and subjected to electrophoresis on a 0.8% agarose gel prepared with Tris-acetate-EDTA (TAE) buffer (40 mM Tris-acetate, 1 mM EDTA). Electrophoresis was carried out at 85 V for 30 min, and the gel was run in TAE buffer. DNA bands were visualized using a Gel Doc XR system (Bio-Rad, Hercules, CA, USA) under UV illumination. The SF (supercoiled form) and NF (nicked form) bands refer to the different conformations of plasmid DNA, where SF represents intact supercoiled plasmid DNA and NF indicates the linear or nicked forms resulting from oxidative cleavage. DNA band intensity was quantified using Image Lab software (version 6.1; Bio-Rad, Hercules, CA, USA), and the percentage of DNA protection was calculated using the following equation with a slight modification:

DNA protection (%) = (Intensity of SF DNA band/Intensity of pUC18 control band) × 100(4)

### 2.10. Statistical Analysis and Data Processing

All quantitative data, including phenolic contents, tyrosinase inhibition, antioxidant activities, TPC, and TFC, are expressed as the mean ± standard deviation (SD) of five independent biological replicates (*n* = 5). Biological replicates were defined as independent batches of dried spinach leaves prepared from separately harvested plant material, each subjected to the full extraction and analytical procedure. No technical replicates were performed; each biological replicate corresponds to a single analytical measurement.

Differences among extraction conditions were analyzed by one-way ANOVA followed by Tukey’s multiple range test at a significance level of *p* < 0.05, performed with SAS 9.2 (SAS Institute Inc., Cary, NC, USA). Multivariate analyses were conducted using R version 4.3.3 (R Project for Statistical Computing, Vienna, Austria). Principal component analysis (PCA) was performed using the ‘prcomp’ function with mean-centering and unit-variance scaling and to calculate principal components for the extraction dataset. The PCA plot, integrating both scores and loadings, was visualized using the ‘ggplot2’ package (version 3.4.4; https://ggplot2.tidyverse.org) to clearly represent the distribution and grouping of extraction conditions. For hierarchical clustering heatmaps, all raw data were standardized using Z-score normalization, calculated as follows:

Z-score = (x − μ)/σ(5)

where x is the individual measurement, μ is the mean of the dataset, and σ is the SD.

This normalization ensured that all variables contributed equally to the clustering result regardless of their absolute scales. Hierarchical clustering analysis was performed on Z-score-normalized data using the Pearson correlation distance and Ward’s linkage method. The resulting heatmaps with dendrograms were generated with the ‘pheatmap’ package in R to visualize the effects of solvent type and methanol concentration on the extraction pattern of the target phenolic phytochemicals [34].

## 3. Results

### 3.1. Evaluation of TPC, TFC, and Biofunctional Properties Using Various Solvents from the Leaves of Winter Spinach

**Figure 1:**
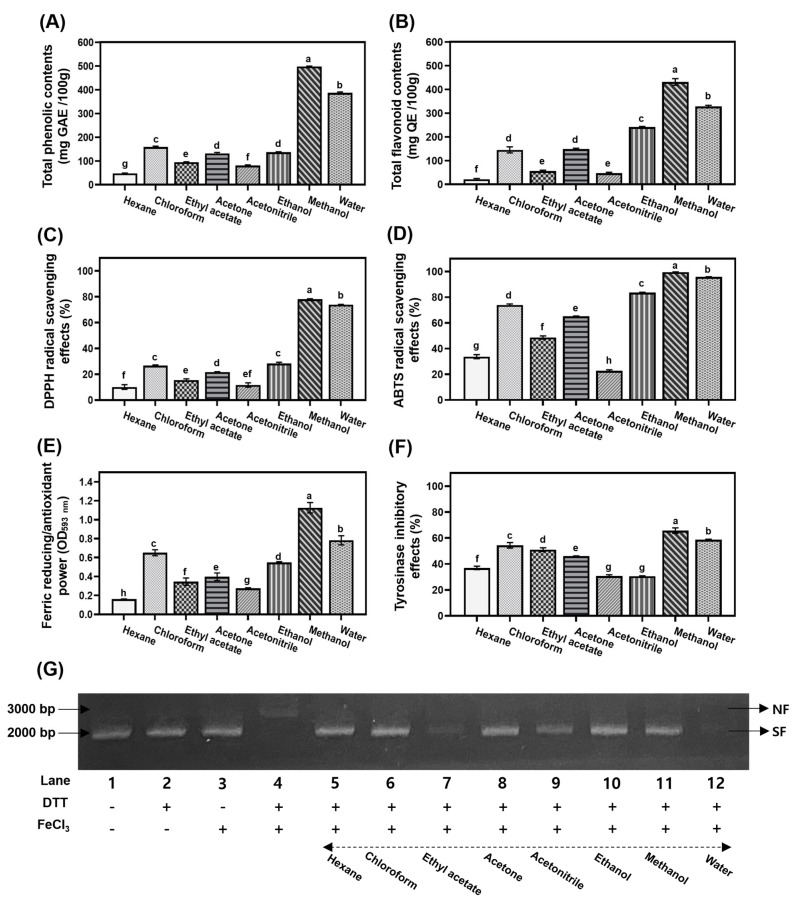

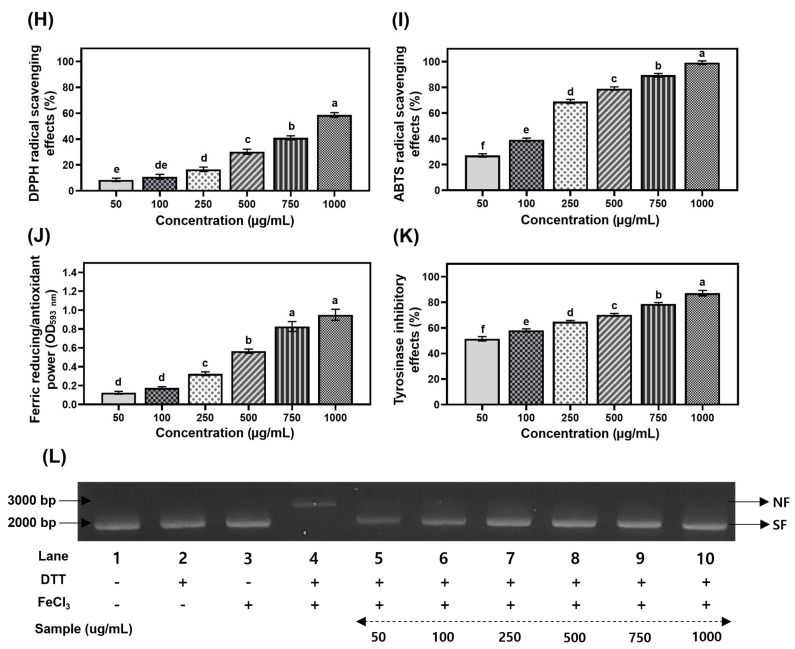
Comparisons of total phenolic content (TPC), total flavonoid content (TFC), and antioxidant properties in different solvent extracts (1000 μg/mL) of winter spinach leaves: (**A**) TPC; (**B**) TFC; (**C**) DPPH radical scavenging activities; (**D**) ABTS radical scavenging activities; (**E**) FRAP; (**F**) tyrosinase inhibitory activities; (**G**) DNA protectant effects: Lane 1, pUC18 only; lane 2, pUC18 with DTT only; lane 3, pUC18 with FeCl_3_ only; lane 4, pUC18 with MCO system; lane 5–12, pUC18 with combinant extracts in the MCO system (lane 5: hexane, lane 6: chloroform, lane 7: ethyl acetate, lane 8: acetone, lane 9: acetonitrile, lane 10: ethanol, lane 11: methanol, and lane 12: water). Comparisons of antioxidant and tyrosinase inhibitory properties in different concentrations of the 50% methanol at 35 °C for 72 h from winter spinach leaves: (**H**) DPPH radical scavenging activities; (**I**) ABTS radical scavenging activities; (**J**) FRAP; (**K**) tyrosinase inhibitory activities; (**L**) DNA protectant effects: Lane 1, pUC18 only; lane 2, pUC18 with DTT only; lane 3, pUC18 with FeCl_3_ only; lane 4, pUC18 with MCO system; lane 5–10, pUC18 with combinant extracts in the MCO system (lane 5: 50 μg/mL, lane 6: 100 μg/mL, lane 7: 250 μg/mL, lane 8: 500 μg/mL, lane 9: 750 μg/mL, lane 10: 1000 μg/mL). Different letters correspond to the significant differences relating to the extraction conditions using Tukey’s multiple range test (*p* < 0.05).

TPC and TFC in spinach leaves were assessed using various solvents, with a focus on their antioxidant and tyrosinase inhibition abilities. The results revealed significant differences in solvents, and their rankings were similar to the observed bioactivities (Figure 1). In TPC patterns, the methanol extract exhibited the most predominant ratio, with 498.0 mg GAE/100 g, while the lowest content was found in the hexane extract at 18.3 mg GAE/100 g (Figure 1A). The second highest ratio was detected in the water extract at 387.1 mg GAE/100 g, followed by chloroform (158.7 mg GAE/100 g) > ethanol (147.1 mg GAE/100 g) > acetone (131.8 mg GAE/100 g) > ethyl acetate (95.2 mg GAE/100 g) > acetonitrile (80.9 mg GAE/100 g) (Figure 1A). The TFC patterns varied significantly depending on the extraction solvents, showing trends similar to those observed for TPC (Figure 1B). The methanol extract yielded the highest level, measuring 311.3 mg QE/100 g, compared to other extracts. The rank order of the other extracts was as follows: water (228.1 mg QE/100 g) > ethanol (131.8 mg QE/100 g) > acetone (108.4 mg QE/100 g) > chloroform (95.6 mg QE/100 g) > ethyl acetate (56.4 mg QE/100 g) > acetonitrile (46.9 mg QE/100 g) > hexane (11.8 mg QE/100 g) (Figure 1B). Preliminary experiments on TPC and TFC analyses were conducted at a concentration of 1000 μg/mL. As illustrated in Figure 1C–E, the individual solvent extracts revealed remarkable differences in antioxidant capacities in radical and FRAP techniques. In the DPPH radical assay, the highest scavenging capacity was found in the methanol extract (78.1%), followed by water (73.9%), while the other solvents showed values below 30% (Figure 1C). The scavenging abilities against ABTS radicals followed the order: 99.5% (methanol) > 95.7% (water) > 83.6% (ethanol) > 73.9% (chloroform) > 65.0% (acetone) > 48.6% (ethyl acetate) > 33.7% (hexane) > 22.6% (acetonitrile) (Figure 1D). The FRAP values followed the same pattern as the radical scavenging assays, with the methanol extract showing the highest value (1.13 OD_593nm_) (Figure 1E). DNA protection rates also differed significantly across solvents, with methanol (98.1%) showing the strongest activity (Figure 1G).

Furthermore, the methanol and water extracts displayed potent tyrosinase inhibitory properties with substantial variations, ranging from 30.5 to 65.7% in different solvents (Figure 1F). Tyrosinase inhibition was strongest in the methanol extract (65.7%), followed by water (58.7%) (Figure 1F). Following optimization, extraction with 50% methanol at 35 °C for 72 h was established as the optimal condition. As a result, the radical scavenging activities exhibited the following concentration-dependent trends (50 → 1000 μg/mL): DPPH: 8.5 → 10.9 → 16.6 → 30.2 → 41.0 → 58.7%, and ABTS: 27.1 → 39.4 → 69.1 → 79.1 → 89.4 → 99.2% (Figure 1H,I). The FRAP values occurred in the following order: 0.95 OD_593nm_ (1000 μg/mL) > 0.83 OD_593nm_ (750 μg/mL) > 0.56 OD_593nm_ (500 μg/mL) > 0.33 OD_593nm_ (250 μg/mL) > 0.18 OD_593nm_ (100 μg/mL) > 0.12 OD_593nm_ (50 μg/mL) (Figure 1J). Tyrosinase inhibitory properties displayed rank order as follows: 1000 μg/mL (87.2%) > 750 μg/mL (78.8%) > 500 μg/mL (70.1%) > 250 μg/mL (64.8%) > 100 μg/mL (58.1%) > 50 μg/mL (51.5%) (Figure 1K). The 50% methanol extract protected DNA from oxidative damage completely (100%) at ≥500 μg/mL, with protection rates progressively decreasing at lower concentrations (Figure 1L). To the best of our knowledge, this study is the first to report variations in biofunctional properties in different extracts of spinach leaves and to establish optimal extraction conditions for phenolic phytochemicals.

### 3.2. Identification of Phenolic Derivatives Using UPLC-Q-TOF-MS/MS Analysis from the Leaves of Winter Spinach

**Figure 2:**
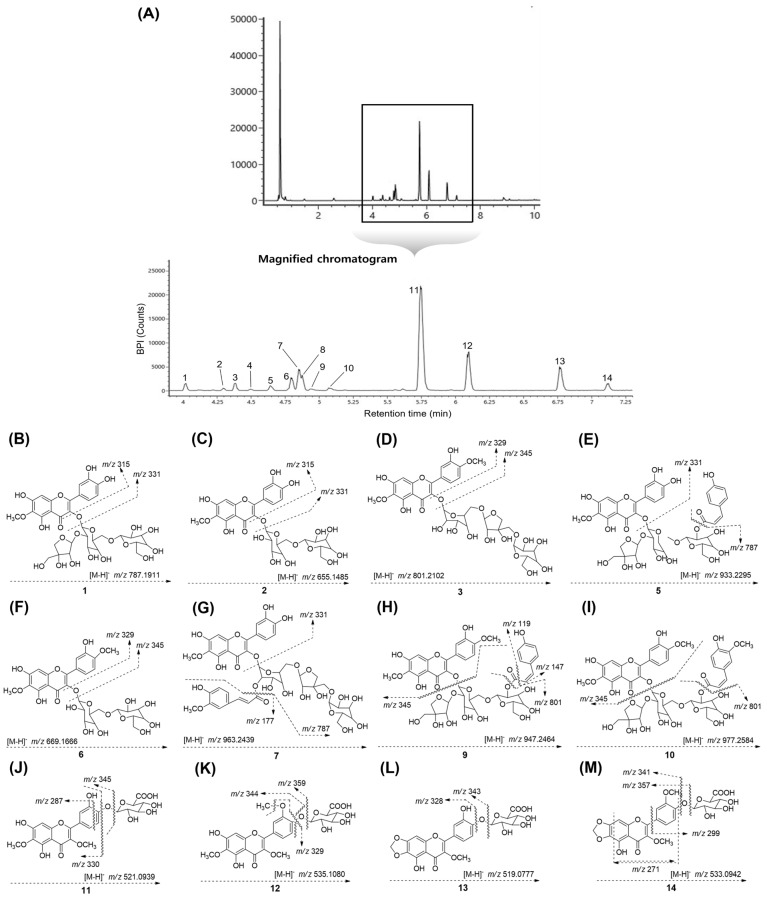
UPLC-Q-TOF-MS chromatogram through negative ion mode of the 50% methanol extract in winter spinach leaves and fragmentation patterns of 12 phenolic derivatives: (**A**) UPLC-Q-TOF-MS chromatogram; (**B**) Mass fragmentation pattern of phenolic **1**; (**C**) Mass fragmentation pattern of phenolic **2**; (**D**) Mass fragmentation pattern of phenolic **3**; (**E**) Mass fragmentation pattern of phenolic **5**; (**F**) Mass fragmentation pattern of phenolic **6**; (**G**) Mass fragmentation pattern of phenolic **7**; (**H**) Mass fragmentation pattern of phenolic **9**; (**I**) Mass fragmentation pattern of phenolic **10**; (**J**) Mass fragmentation pattern of phenolic **11**; (**K**) Mass fragmentation pattern of phenolic **12**; (**L**) Mass fragmentation pattern of phenolic **13**; (**M**) Mass fragmentation pattern of phenolic **14**.

To ensure transparency in phenolic identification, the phenolic constituents were categorized into three confidence levels: putatively identified phenolics, assigned based on accurate mass measurements, MS/MS fragmentation patterns, and comparison with previously reported spinach metabolite data (phenolics **1**, **2**, **3**, **5**, **6**, **7**, **9**, **10**, and **14**; Figures S1–S12); confirmed phenolics, isolated and structurally elucidated by 1D/2D NMR spectroscopy in combination with UPLC-Q-TOF-MS/MS (phenolics **11**, **12**, and **13**; Figures S13–S20); and tentatively identified phenolics, assigned as structural isomers based on identical molecular masses and similar fragment ion patterns (phenolics **4** and **8**). **14** phenolic derivatives of spinach leaves were systematically characterized using UPLC-Q-TOF-MS/MS (Figure 2A). Structural elucidation was conducted systematically for each compound, based on the interpretation of high-resolution mass spectrometric data, chromatographic retention times, and characteristic fragmentation patterns observed under negative ion electrospray ionization mode (Table S1). Comparative analysis with reported literature and databases. As illustrated in Figure 2A, the representative BPI chromatogram achieved complete separation of diverse metabolites, including both major and minor peaks, within 8 min. Three major phenolics (**11**–**13**) constituted approximately 70% of the total peak area, while the remaining peaks (**1**–**10** and **14**) accounted for about 30%. Herein, their structural characteristics were documented with full scan negative ion MS and MS^2^ spectra (Table 1 and Figure 2B–M). The identified 12 phenolics, including several conjugated forms such as glucuronides, feruloyl, and coumaroyl conjugates, as well as di- and triglycosides, were detected. These flavonoids were found to possess patuletin, spinacetin, spinatoside, jaceidin, and flavone as their aglycone moieties, in agreement with previous studies [8,12,25,30,31]. The following information provides detailed characterization of individual compounds as identified by the TOF-MS/MS analysis.

The UPLC-TOF mass spectrum of peak 1 (*t_R_* = 4.02 min; mass error: 0.258 ppm) showed the molecular ion [M–H]^−^ at *m*/*z* 787.1911. The fragmentation ions of the MS/MS spectrum were detected at signals at *m*/*z* 331.0420 ([M–H]^−^–56 amu) and 315.0126 ([M–H]^−^–472 amu) (Figure 2B). The fragment ion (*m*/*z* 331.0420) was characteristic of the patuletin aglycone, formed through the loss of the deprotonated sugar moieties {1 apiose (132 amu, C_5_H_10_O_5_) and 2 glucose (2 × 162 amu, 2 × C_6_H_12_O_6_) units} [8]. Additionally, the remaining MS/MS fragment ion (*m*/*z* 315.0126) was observed as the loss of the hydroxyl group (17 amu-H^+^) in the patuletin aglycone (*m*/*z* 331.0420) (Figure 2B) [6,33]. Based on these observations and previous literature, peak 1 was identified as patuletin-3-*O*-β-D-glucopyranosyl-(1→6)-[β-D-apiofuranosyl-(1→2)]-β-D-glucopyranoside (C_33_H_40_O_22_) [8,25]. Full TOF mass scan analysis of peak 2 (*t_R_* = 4.29 min; mass error: −0.545 ppm) showed the [M–H]^−^ ion at *m*/*z* 655.1485, with two fragmentation ions at *m*/*z* 331.0443 and 315.0126 (Figure 2C). Similar to peak 1, its MS/MS spectrum generated a characteristic fragment ion at *m*/*z* 331.0443, confirming the presence of the patuletin aglycone (C_16_H_12_O_8_) [8,28]. Thus, the major fragmentation at *m*/*z* 331.0443 indicated the loss of two hexose units (*m*/*z* 655 {[M–H]^−^} → *m*/*z* 324 (2 × 162 amu; 2 × glucose)). Also, the fragment ion at *m*/*z* 315.0126 in the MS/MS spectrum was formed by the loss of an OH group (*m*/*z* 16; *m*/*z* 331 → *m*/*z* 315; ({[M–H]^−^–OH)–H^+^}) (Figure 2C) [6,33]. On the basis of these considerations and published data, peak 2 was tentatively identified as patuletin-3-*O*-β-D-glucopyranosyl-(1→6)-β-D-glucopyranoside (C_28_H_32_O_18_) [12,28,30]. The fragmentation patterns of peaks 5 (*m*/*z* 331.0420) and 7 (*m*/*z* 331.0421) were similar to those of peaks 1 (*m*/*z* 331.0420) and 2 (*m*/*z* 331.0443); in particular, the fragmentation at *m*/*z* 331 was assigned to the patuletin aglycone by comparison with previously reported data [8,28]. In more detail, peak 5 (*t_R_* = 4.64 min; mass error: −0.616 ppm) presented a deprotonated molecular ion [M–H]^−^ at *m*/*z* 933.2295 and two fragment ions at *m*/*z* 787.1946 and *m*/*z* 331.0420 (Figure 2E). The major fragmentation at *m*/*z* 787.1946 (−146 amu) resulted from the loss of a coumaroyl moiety (C_9_H_8_O_2_) [8,35], while the minor ion at *m*/*z* 331.0420 was determined to be characteristic of patuletin aglycone residue by comparing the data reported in earlier literature [28]. as well as those of peaks 1 and 2. Therefore, it was tentatively identified as patuletin-3-*O*-β-D-(2″-β-coumaroylglucopyranosyl)-(1→6)-[β-D-apiofuranosyl-(1→2)]-β-D-glucopyranoside (**5**) (C_42_H_46_O_24_) [25,31]. Subsequently, the TOF-MS in negative ion mode of peak 7 (*t_R_* = 4.85 min; mass error: −0.746 ppm) exhibited the deprotonated precursor [M–H]^−^ at *m*/*z* 963.2406. Moreover, three fragment ions at *m*/*z* 787.1946, *m*/*z* 331.0365, and *m*/*z* 177.1600 were similar to those of peak 5 (Figure 2G). Among these fragment ions, the product ion peak at *m*/*z* 787.1946 {([M–H]^−^–177 amu)–H^+^} was potentially formed by the loss of the feruloyl moiety (177 amu; C_10_H_10_O_4_) [8,35]. The remaining ions, such as *m*/*z* 331.0365 and *m*/*z* 177.1600, displayed a deprotonated patuletin aglycone {[M–H]^−^–(456 amu: 1 apiose (132 amu) and two glucose (2 × 162 amu) + 177 amu)–H^+^} and a ferulonyl residue ([M–H]^−^–787 amu) (Figure 2G) [12,25]. Based on the accurate mass values and earlier literature reports, the current peak was tentatively assigned to patuletin 3-*O*-(2″-feruloylglucosyl)-(1→6)-[apiofuranosyl-(1→2)]-glucopyranoside (**7**) (C_43_H_48_O_25_) [31,35]. The TOF-MS profile of peak 3 (*t_R_* = 4.38 min; mass error: 0.253 ppm) showed the deprotonated molecular ion [M–H]^−^ at *m*/*z* 801.2102 and the MS/MS fragment (*m*/*z* 345.0594) resulted from the loss of a complex sugar group (one apiose: *m*/*z* 132 amu and two glucoses: 2 × 162 amu), corresponding to the molecular ion of the spinacetin aglycone (C_17_H_14_O_8_) (Figure 2D) [26,29]. Another MS/MS fragment ion at *m*/*z* 329.0280 was generated through the loss of a hydroxyl moiety (17 amu–H) from the parent ion at *m*/*z* 345 of the spinacetin aglycone. Based on the evidence presented above and earlier reports, peak 3 was tentatively evaluated as spinacetin-3-*O*-β-D-glucopyranosyl-(1→6)-[apiofuranosyl-(1→2)]-β-D-glucopyranoside (**3**) (C_34_H_42_O_22_) [12,28,29]. Peak 6 (*t_R_* = 4.79 min; mass error: −0.519 ppm) displayed an identical molecular ion [M–H]^−^ at *m*/*z* 669.1666. The MS/MS spectrum exhibited two fragment ions, including one major ion at *m*/*z* 345.0594 and one minor ion at *m*/*z* 329.0280 (Figure 2F). These two fragment ions were consistent with the ion patterns of peak 2 [30,31]. The above molecular weight may be attributed to the flavonoid aglycone characteristics, (*m*/*z* 669.1672), corresponding to the spinacetin structure, by comparison with earlier reported spinach phytochemicals [8,30]. The predominant fragment ion at *m*/*z* 345.0594 was generated by the sequential loss of two glucose moieties (2 × 162 amu) from the deprotonated molecular ion (*m*/*z* 669.1666), indicating the presence of two glucose residues. Additionally, the fragment ion at *m*/*z* 329.0280 resulted from the elimination of an –OH radical, yielding the ion (([M–H]^−^–2 × glucose)–OH)^−^. According to the summarized fragmentation and the published results, peak 6 was confirmed to be spinacetin-3-*O*-β-D-glucopyranosyl-(1→6)-β-D-glucopyranoside (**6**) with the elemental formula C_29_H_34_O_18_ [30,31]. product ions in UPLC-TOF-MS spectra of peaks 9 (*t_R_* = 4.94 min; mass error: 0.710 ppm) and 10 (*t_R_* = 5.07 min; mass error: 0.857 ppm) displayed [M–H]^−^ ions at *m*/*z* 947.2464 and *m*/*z* 977.2584, respectively, and their characteristic fragment ions were observed at *m*/*z* 801.2066, *m*/*z* 345.0594, and *m*/*z* 119.0503 for peak 9 and *m*/*z* 801.2102 and *m*/*z* 315.0517 for peak 10 (Figure 2H,I). Both peaks exhibited highly similar ionization patterns based on their fragmentation files. In the MS/MS spectrum of peak 9, the fragment ion at *m*/*z* 119.0503 corresponds to the coumaric acid moiety, indicating the presence of a coumaroyl group (147 amu–H) [8,35]. The ion at *m*/*z* 345.0594 was assigned to a spinacetin structure through the combined losses of deprotonated sugar moieties (1 apiose: 132 amu and 2 glucose: 2 × 162 amu) and a coumaroyl unit (147 amu–H) (Figure 2H) [8,25]. These fragmentation patterns were similar to those of peak 5. Moreover, the major fragmentation at *m*/*z* 801.2066 was attributed to the neutral loss of *m*/*z* 146 amu (coumaroyl group: 147 amu–H) from the precursor ion (*m*/*z* 947.2464) (Figure 2H). Based on the earlier literature on spinach and the above fragmentation evidence, this peak was identified as spinacetin-3-*O*-β-D-(2″-β-coumaroylglucopyranosyl)-(1→6)-[β-D-apiofuranosyl-(1→2)]-β-D-glucopyranoside (**9**) with the formula C_43_H_48_O_24_ [25,31]. In the MS/MS spectrum of peak 10, the fragment ion at *m*/*z* 345.0517 was formed by the loss of the *m*/*z* 632 (*m*/*z* 977 → *m*/*z* 345). This molecular weight was characteristic of the aglycone, specifically the spinacetin structure, as supported by comparison with peaks 3, 6, and 9 and published studies [29,35]. Furthermore, this fragment ion may result from the losses of a sugar group (456 amu: 1 apiose: 132 amu + 2 glucose: 2 × 162 amu) and a ferulonyl unit (176 amu) (Figure 2I) [8,35]. Especially, the fragment ion at *m*/*z* 801.2102 corresponds to the loss of a feruloyl group (977–176 amu), confirming the presence of a feruloyl substitution instead of a coumaroyl group (146 amu) in peak 9, in agreement with previous research [8,35]. Thus, this peak was tentatively assigned as spinacetin-3-*O*-β-D-(2″feruloylglucopyranosyl) (1→6)-[β-D-apiofuranosyl(1→2)]-β-D-glucopyranoside (**10**) (C_44_H_50_O_23_) [12,28]. The full mass scan analysis of peak 11 (*t_R_* = 5.74 min; mass error: −0.649 ppm) was observed with a deprotonated molecular ion [M–H]^−^ at *m*/*z* 521.0939 and three fragment ions at *m*/*z* 345.0617, *m*/*z* 330.0365, and *m*/*z* 315.0126 (Figure 2J). The product ion at *m*/*z* 345.0617 was potentially formed by the loss of the glucuronide moiety (176 amu) [26]. In addition, the fragment ion at *m*/*z* 330.0365 corresponded to the deprotonated spinacetin aglycone (C_17_H_14_O_8_), resulting from the loss of a glucuronic acid unit (C_6_H_10_O_7_, 192 amu), consistent with previous research on spinach [26,28]. In other words, the loss of 191 amu from [M–H]^−^ can represent the complete glucuronic acid residue with proton transfer [26,34]. The other two fragment ions at *m*/*z* 330.0365 and *m*/*z* 315.0126 corresponded to the losses of one methyl group (345 amu–15 amu) and two methyl groups (345 amu–2 × 15 amu) from the product ion at *m*/*z* 345.0617, as supported by previous reports [12,28]. Therefore, peak 11 was identified as spinatoside (C_23_H_22_O_14_), characterized as a spinacetin aglycone conjugated with glucuronic acid [28,30]. The MS analysis of peak 12 (*t_R_* = 6.10 min; mass error: −0.128 ppm) revealed a deprotonated molecular ion [M–H]^−^ ions at *m*/*z* 535.1080, with fragment ions at *m*/*z* 359.0765, 344.0517, and 329.0280 observed in the MS/MS spectrum (Figure 2K). The characteristic product ion at *m*/*z* 359.0765 was formed by the loss of a *m*/*z* 176 amu fragment (*m*/*z* 535 → *m*/*z* 359; [M–H]^−^–176 amu), indicating the presence of a glucuronide moiety [8,35]. Furthermore, this ion corresponds to the deprotonated jaceidin aglycone (C_16_H_12_O_6_, 360 amu) by comparison with the previously reported data [26,28]. A fragment ion at *m*/*z* 344.0157 was observed as the result of loss of methyl and glucuronide groups ([M–H]^−^–(176 amu + 15 amu)), in agreement with earlier studies [28,33], and the signal at *m*/*z* 329.0280 was attributed to the loss of a methyl moiety ([M–H]^−^–(176 amu + 15 amu)–(15 amu)) from the ion at *m*/*z* 344.0517 [28]. According to the summarized ions and spinach research information, peak 12 was elucidated as jaceidin-4′-β-D-glucuronide (**12**) (C_24_H_24_O_14_) [12,30]. The TOF-MS and MS/MS spectra of peak 13 (*t_R_* = 6.76 min; mass error: 0.694 ppm) showed an [M–H]^−^ ion at *m*/*z* 519.0777 and two fragment ions at *m*/*z* 343.0455 and *m*/*z* 328.0211 (Figure 2L). The major fragmentation at *m*/*z* 343.0455 (−176 amu) may be the result of the loss of a glucuronic moiety (519 amu–176 amu), similar to those observed in peaks 11 and 12. In addition, the remaining ion at 328.0211 was attributed to the loss of glucuronic acid (176 amu) and methyl (15 amu) groups from the deprotonated molecular ion [M–H]^−^ at *m*/*z* 519.0777. From the earlier literature on the spinach plant, this peak was deduced as 5,3′,4′-trihydroxy-3-methoxy-6,7-methylenedioxyflavone-4′-β-D-glucuronide (**13**) (C_23_H_20_O_14_) [12,25,30]. The deprotonated molecular ion [M–H]^−^ at *m*/*z* 533.0942 was found in the UPLC-TOF-MS spectrum of peak 14 (*t_R_* = 7.12 min; mass error: 0.638 ppm), and three fragment ions (*m*/*z* 357.0604, *m*/*z* 341.0360, and *m*/*z* 299.0189) were detected in the MS/MS data (Figure 2M). The ion at *m*/*z* 357.0604 showed a fragmentation pattern similar to those observed in peaks 11–13. The fragment ion at *m*/*z* 341.0360 was attributed to the loss of *m*/*z* 192 amu ([M–H]^−^–(176 amu: glucuronic acid + 18 amu: H_2_O)–2H) as the neutral loss of glucuronic acid with dehydration [8,26]. The ion at *m*/*z* 299.0189 may likely be generated by the cleavage of the glucuronic group (176 amu) and partial ring fragmentation (123 amu) [33,36]. By comparing their mass values with data reported in the earlier literature, this peak was tentatively assigned as 5,4′-dihydroxy-3,3′-dimethoxy-6,7-methylenedioxyflavone-4′-β-D-glucuronide (**14**) (C_24_H_22_O_14_) [12,25,28,30]. Furthermore, peaks 4 and 8 were elucidated as structural isomers corresponding to peaks 3 and 7, based on their identical molecular masses and fragment ion patterns observed in the UPLC-Q-TOF-MS/MS spectra.

### 3.3. NMR Spectroscopic Data of Three Major Phenolics Isolated from the Leaves of Winter Spinach

The chemical structures of three isolated phenolics **11**–**13** were elucidated by NMR spectroscopy and identified as follows: spinatoside (**11**), jaceidin-4′-β-D-glucuronide (**12**), and 5,3′,4′-trihydroxy-3-methoxy-6,7-methylenedioxyflavone-4′-β-D-glucuronide (**13**). Comprehensive NMR data for these phenolics are presented in the supplementary information (Figures S13–S20). ^1^H and ^13C^ NMR spectroscopic data are detailed below.

#### 3.3.1. Spinatoside (**11**)

Amorphous yellow powder; Mass value [M-H]^−^ *m*/*z* 521.0939 {Calculated mass [M-H]^−^ (*m*/*z*) 521.0936}; ^1^H NMR (500 MHz, CD_3_OD): δ 3.54–3.61 (3H, m, H-2″, H-3″, H-4″: Glu moiety), 3.79 (3H, s, 3-OCH_3_: H-11), 3.87 (3H, s, 6-OCH_3_: H-12), 4.06 (1H, d, *J* = 10.0 Hz, H-5″), 5.03 (1H, d, *J* = 10.0 Hz, H-1″), 6.48 (1H, s, H-8), 7.23 (1H, d, *J* = 10.0 Hz, H-5′), 7.56 (1H, d, *J* = 10.0 Hz, H-6′), and 7.61 (1H, s, H-2′). ^13^C NMR (125 MHz, CD_3_OD): δ 59.27 (3-OCH_3_: C-11), 59.55 (6-OCH_3_: C-12), 71.56 (C-4″), 73.02 (C-3″), 75.21 (C-5″), 75.47 (C-2″), 93.64 (C-8), 101.73 (C-1″), 105.01 (C-10), 115.85 (C-2′), 116.25 (C-5′), 120.47 (C-6′), 125.30 (C-1′), 131.15 (C-6), 138.40 (C-3), 146.74 (C-3′), 147.35 (C-4′), 152.24 (C-5), 152.32 (C-2), 155.69 (C-7), 157.41 (C-9), 170.76 (C-6″), and 178.90 (C-4).

#### 3.3.2. Jaceidin-4′-β-D-glucuronide (**12**)

Amorphous pale yellow powder; Mass value [M-H]^−^ *m*/*z* 535.1080 {Calculated mass [M-H]^−^ (*m*/*z*) 535.1093}; ^1^H NMR (500 MHz, CD_3_OD): δ 3.52–3.67 (3H, m, H-2″, H-3″, H-4″: Glu moiety), 3.81 (3H, s, 3-OCH_3_: H-11), 3.88 (3H, s, 6-OCH_3_: H-12), 4.03 (1H, d, *J* = 10.0 Hz, H-5″), 5.14 (1H, d, *J* = 10.0 Hz, H-1″), 6.53 (1H, s, H-8), 7.26 (1H, d, *J* = 10.0 Hz, H-5′), 7.69 (1H, d, *J* = 10.0 Hz, H-6′), and 7.74 (1H, s, H-2′). ^13^C NMR (125 MHz, CD_3_OD): δ 56.85 (3′-OCH_3_: C-13), 60.76 (3-OCH_3_: C-11), 60.97 (6-OCH_3_: C-12), 72.92 (C-4″), 74.50 (C-3″), 76.64 (C-5″), 77.24 (C-2″), 95.14 (C-8), 102.01 (C-1″), 106.47 (C-10), 113.68 (C-2′), 117.25 (C-5′), 123.20 (C-6′), 126.23 (C-1′), 132.63 (C-6), 139.84 (C-3), 150.06 (C-3′), 150.73 (C-4′), 153.72 (C-5), 153.82 (C-2), 157.16 (C-7), 158.90 (C-9), 172.12 (C-6″), and 180.35 (C-4).

#### 3.3.3. 5,3′,4′-trihydroxy-3-methoxy-6,7-methylenedioxyflavone-4′-β-D-glucuronide (**13**)

Amorphous yellow powder; Mass value [M-H]^−^ *m*/*z* 519.0777 {Calculated mass [M-H]^−^ (*m*/*z*) 519.0780}; ^1^H NMR (500 MHz, CD_3_OD): δ 3.54–3.68 (3H, m, H-2″, H-3″, H-4″: Glu moiety), 3.80 (3H, s, 3-OCH_3_: H-11), 4.06 (1H, d, *J* = 10.0 Hz, H-5″), 5.04 (1H, d, *J* = 10.0 Hz, H-1″), 6.08 (2H, s, H-12), 6.63 (1H, s, H-8), 7.23 (1H, d, *J* = 10.0 Hz, H-5′), 7.56 (1H, dd, *J* = 10, 5.0 Hz, H-6′), and 7.60 (1H, s, H-2′). ^13^C NMR (125 MHz, CD_3_OD): δ 60.24 (3-OCH_3_: C-11), 71.86 (C-4″), 73.34 (C-2″), 75.73 (C-5″), 75.80 (C-3″), 90.12 (C-8), 100.91 (C-1″), 103.31 (C-12), 107.72 (C-10), 115.94 (C-2′), 116.22 (C-5′), 120.58 (C-6′), 124.38 (C-1′), 129.70 (C-6), 138.68 (C-3), 140.84 (C-3′), 147.03 (C-4′), 147.79 (C-5), 152.26 (C-2), 154.47 (C-7), 155.78 (C-9), 170.62 (C-6″), and 179.00 (C-4).

### 3.4. Determination of Three Major Phenolics in the Leaves of Winter Spinach Using Optimized Extraction Conditions

**Figure 3:**
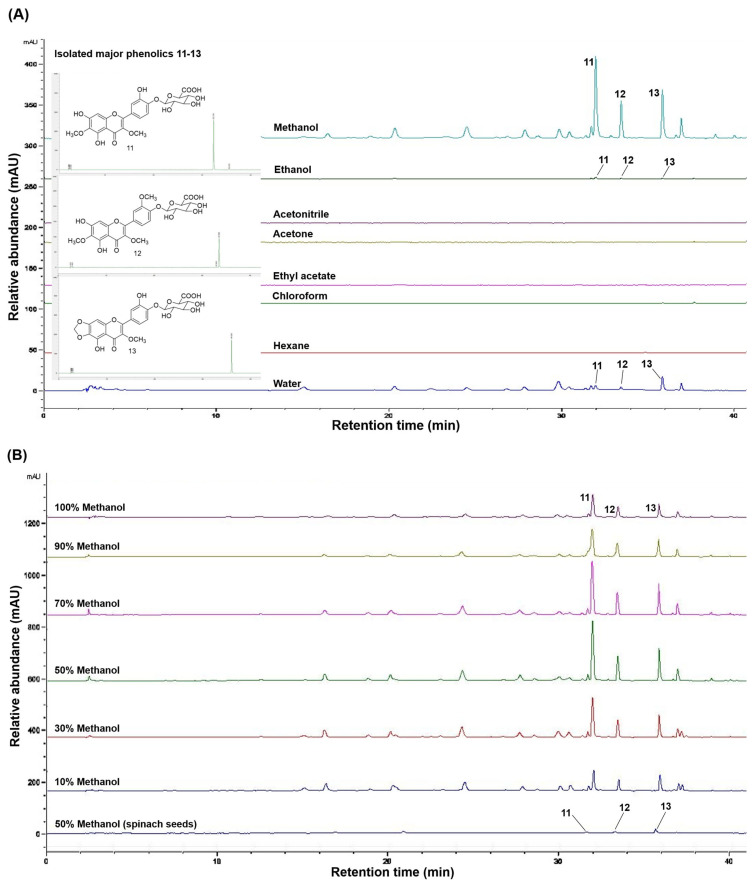
Comparison of HPLC chromatograms concern to three major phenolic phytochemicals **11**–**13**: (**A**) Different solvent extracts using winter spinach leaves; (**B**) Different extracts of methanol and water mixtures using winter spinach leaves (10, 30, 50, 70, 90, and 100% methanol) and seeds (50% methanol).

As a first step, we systematically evaluated the extraction efficiency of major phenolics using diverse solvents with differing polarity characteristics at room temperature for 24 h. The retention times of three phenolics were detected in the HPLC chromatogram at 330 nm as follows: peak 11 (*t_R_* = 32.3 min), peak 12 (*t_R_* = 33.7 min), and peak 13 (*t_R_* = 35.9 min) (Figure 3A). As illustrated in Figure 3A, hexane, chloroform, ethyl acetate, acetone, and acetonitrile extracts did not show detectable levels of the three major phenolics. The methanol extract yielded the most abundant content of major phenolics, at 2716.2 μg/g (**11**: 1352.5 μg/g, **12**: 653.2 μg/g, and **13**: 710.5 μg/g), while the water extract had a total of 218.3 μg/g (**11**: 58.1 μg/g, **12**: 6.0 μg/g, and **13**: 154.2 μg/g) (Figure 3A and Table 2). In the ethanol extract, only phenolic **11** exhibited a minor content of 20.3 μg/g, while phenolics **12** and **13** were present in trace amounts, indicating extremely low abundance.

To further investigate extraction efficiency, various extraction times (12, 24, 36, 48, 60, and 72 h) and temperatures (25, 35, and 45 °C) were analyzed. Although individual experiments revealed only mild differences depending on extraction conditions, the optimal extraction for major phenolics in spinach leaves was achieved using methanol for 72 h at 35 °C. To optimize extraction efficiency using solvent-water mixtures, we analyzed phenolic amounts at various water to methanol ratios (10, 30, 50, 70, 90, and 100%) for 72 h at 35 °C, as illustrated in Figure 3B. As shown in Table 2, the extraction efficiency of the three major phenolics (**11**–**13**) was markedly influenced by the methanol concentration in the aqueous solvent system. In other words, extraction efficiency varied significantly with the methanol ratio (Figure 3B). At 10% methanol, all three phenolics were detected, yielding a total content of 2514.3 μg/g (**11**: 1130.3 μg/g, **12**: 674.7 μg/g, and **13**: 709.3 μg/g), indicating measurable extraction even at a low methanol concentration. As the methanol concentration increased to 30%, the yields rose substantially, with **11** at 2170.2 μg/g, **12** at 1026.4 μg/g, and **13** at 1111.8 μg/g, totaling 4308.4 μg/g. The maximum extraction efficiency was achieved at 50% methanol, yielding 6525.6 μg/g (**11**: 3366.2 μg/g, **12**: 1532.5 μg/g, and **13**: 1626.9 μg/g). This suggests that 50% methanol provides optimal solvent polarity for recovering the three major phenolics. As methanol concentration increased, all three phenolic contents significantly increased up to 50% methanol (2514.3 → 4308.4 → 6525.6 μg/g). However, the 70% methanol extract yielded 6261.5 μg/g, which was lower than that of the 50% methanol extract (6525.6 μg/g) (Table 2). This phenomenon may be attributed to the increased flow resistance of 70% methanol, which restricts solvent penetration into plant cell walls, and the potential adsorption of phenolic compounds at higher concentrations [14,20,26]. Additionally, higher methanol extracts at 90 and 100% showed a gradual decline in yield, with totals of 4385.2 μg/g (90% methanol) and 2879.0 μg/g (100% methanol). Specifically, the individual phenolics in the range of 70 to 100% methanol decreased as follows: **11**: 3237.1 → 2181.4 → 1427.0 μg/g; **12**: 1488.6 → 985.3 → 697.6 μg/g; and **13**: 1535.9 → 1118.5 → 754.4 μg/g (Table 2).

Our results clearly indicate that the extraction efficiency of the three major phenolics is strongly influenced by the polarity of the solvent system, with 50% methanol in water providing the highest yield under the given extraction conditions. These findings are consistent with the existing literature, emphasizing that systematic optimization of extraction conditions is key to enhancing phenolic yield. Previous research has also reported that mixed solvent systems with intermediate polarity are optimal for extracting phenolic phytochemicals from natural materials and edible plants [9,15,20,26]. Interestingly, under the optimal conditions outlined above, the three major phenolics in spinach seeds were identified as **11** (118.3 μg/g), **12** (130.6 μg/g), and **13** (210.2 μg/g), with a total content of 459.1 μg/g (Figure 3B and Table 2). This amount is significantly lower than phenolic contents observed in the leaves. Therefore, our work reveals that spinach leaves, due to their higher phenolic contents, may be a more effective natural source than seeds for functional food applications and nutraceuticals.

### 3.5. Biofunctional Effects of Three Major Phenolics Isolated from the Leaves of Winter Spinach

**Figure 4:**
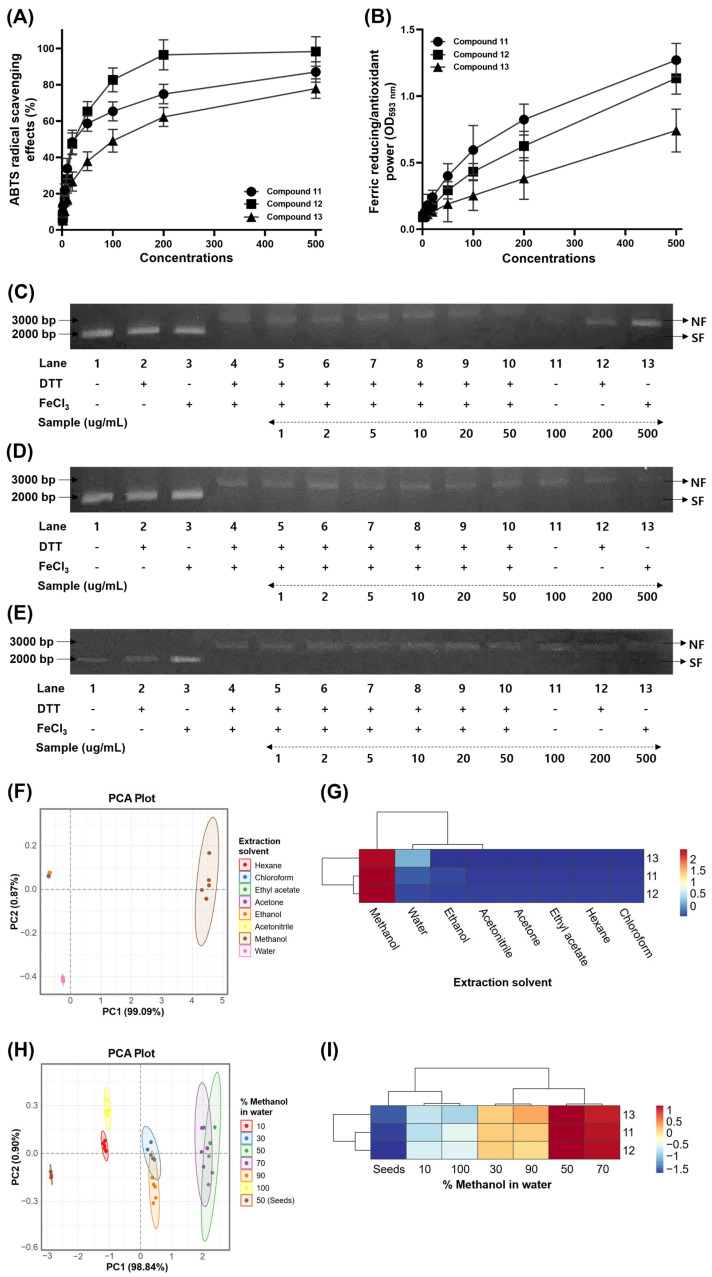
Comparisons of biofunctional effects and principal component analysis (PCA) and heatmap analysis of three major phenolics **11**–**13**: (**A**) ABTS radical scavenging activities; (**B**) FRAP; (**C**) DNA protectant activities of phenolic **11**; (**D**) DNA protectant activities of phenolic **12**; (**E**) DNA protectant activities of phenolic **13**; (**F**) Score plot of PCA in different solvent extracts of winter spinach leaves; (**G**) Hierarchical clustering and heatmap analysis to investigate the variable phenolics’ relationships in different solvent extracts of winter spinach leaves, either positive (red) or negative (blue); (**H**) Score plot of PCA in water-methanol mixture extracts of winter spinach leaves and seeds; (**I**) Hierarchical clustering and heatmap analysis to investigate the variable phenolics’ relationships in water-methanol mixture extracts of winter spinach leaves and seeds, either positive (red) or negative (blue).

To explore more information regarding the biological properties of winter spinach, we measured the radical scavenging activities, FRAP values, tyrosinase inhibitions, and DNA protection ratios of the three major isolated phenolics **11**–**13** (Figure 4A–E). Biofunctional capacities were assessed by diluting individual stock solutions (500 μM) with methanol to create nine different concentrations (500, 200, 100, 50, 20, 10, 5, 2, and 1 μM). The three phenolics demonstrated higher radical scavenging abilities in the ABTS assay compared to the DPPH radical. Specifically, the DPPH radical scavenging effects were approximately 20–30% lower than those observed in the ABTS assay in various concentrations of each compound. The most potent DPPH radical inhibitor was phenolic **12** (IC_50_ = 57.6 μM), followed by phenolic **11** (IC_50_ = 83.7 μM), while phenolic **13** was inactive (IC_50_ > 200 μM); BHT was used as the positive control (IC_50_ = 26.7 μM) [6]. Phenolic **11** showed the second highest scavenging effect on DPPH, with an IC_50_ value of 83.7 μM, while phenolic **13** exhibited a low effect, with an IC_50_ value exceeding 200 μM. The ABTS radical scavenging abilities revealed considerable differences in the phenolics, exhibiting dose-dependent variations. The inhibitions at concentrations from 500 to 1 μM were as follows: **11**: 87.0% at 500 μM, 74.9% at 200 μM, 65.6% at 100 μM, and 58.7% at 50 μM; **12**: 98.3, 96.5, 82.7, and 65.2%; **13**: 77.9, 62.3, 49.1, and 37.9% (Figure 4A). In the ABTS assay, phenolic **12** again showed the strongest activity (IC_50_ = 21.9 μM), followed by phenolic **11** (IC_50_ = 29.5 μM) and phenolic **13** (IC_50_ = 92.2 μM); Trolox was used as the positive control (IC_50_ = 18.7 μM) [6].

To further understand the antioxidant properties of the three phenolics, additional parameters such as FRAP and DNA protection were evaluated (Figure 4B,C). The antioxidant effects in the FRAP assay showed considerable differences in various concentrations (500 → 1 μM) of the three phenolics, with values increasing in the order of phenolics **11** > **12** > **13** (**11**: 1.27 → 0.82 → 0.60 → 0.40 → 0.24 OD_593nm_, 10–1 μM < 0.18 OD_593nm_; **12**: 1.13 → 0.63 → 0.43 → 0.29 OD_593nm_, 20–1 μM < 0.20 OD_593nm_; **13**: 0.74 → 0.38 → 0.25 OD_593nm_, 50–1 μM < 0.20 OD_593nm_) (Figure 4B). DNA protection rates varied in a concentration-dependent manner among the three phenolics. Phenolic **11** showed the highest DNA protection activity, reaching 73.9% at 500 μM, while phenolics **12** and **13** displayed lower protection rates (<40%; Figure 4C–E). In summary, phenolics **11**–**13** demonstrated potent natural antioxidant activities in radical scavenging assays, with their effectiveness decreasing in the following order: **11** > **12** > **13**. To the best of our knowledge, this research represents the first comparative investigation assessing and comparing FRAP, tyrosinase inhibition, radical scavenging activity, and DNA protection rates in the phenolics present in winter spinach leaves.

### 3.6. Correlation Analysis of Three Major Phenolics in the Leaves of Winter Spinach Based on Different Solvent Systems

To explore the interrelationships among the three dominant phenolic derivatives **11**–**13** in winter spinach leaves, multivariate analyses, including PCA and hierarchical clustering, were conducted. These analyses were based on phenolic content data extracted using various organic solvents and aqueous methanol mixtures, following previously reported approaches [19,34]. The hierarchical clustering heatmaps based on standardized z-scores revealed correlation patterns between phenolics and solvents, indicating positive (red) or negative (blue) associations (Figure 4F–I) [19,34]. As shown in Figure 4F, the PCA of solvent-based extraction revealed a clear separation along the first principal component (PC1), which accounted for over 99% of the total variance. This dominance limits the interpretative value of the PCA for mechanistic insights; therefore, it is recommended to consider adding more variables for a more comprehensive analysis. Samples extracted with methanol were distinctly grouped apart from those extracted with other solvents, indicating its superior efficiency. Methanol alone yielded the highest total content of phenolics **11**–**13** (2716.2 µg/g), with individual contributions of phenolic **11** at 1352.5 µg/g, phenolic **12** at 653.2 µg/g, and phenolic **13** at 710.5 µg/g. In contrast, other solvents such as hexane, chloroform, ethyl acetate, and acetonitrile failed to recover detectable amounts of these phenolics (ND), demonstrating the limited solubility of these polar phenolics in non-polar or aprotic environments. Interestingly, ethanol, although polar, extracted only trace levels (20.3 µg/g), primarily phenolic **11**, while water yielded modest levels of phenolic **11** (58.1 µg/g), phenolic **12** (6.0 µg/g), and phenolic **13** (154.2 µg/g), suggesting the hydrophilic nature of some phenolic moieties, especially phenolic **13**. The clustering heatmap (Figure 4G) further supported this interpretation, with methanol forming an isolated cluster and water showing partial similarity. To refine extraction efficiency, a gradient of methanol concentrations (10–100% in water) was employed for 72 h at 35 °C. The PCA (Figure 4H) showed that PC1 (98.84%) and PC2 (0.90%) explained almost all the variance, with optimal phenolic recovery observed at 50–70% methanol (Table 2), indicating strong solubility and matrix penetration at these solvent polarities. Conversely, lower methanol concentrations (10–30%) resulted in substantially reduced yields and formed separate clusters in the PCA and heatmap, reflecting inadequate extraction. Extraction with 100% methanol yielded lower values than those obtained with 50–70%. The hierarchical clustering heatmap (Figure 4I) mirrored the PCA patterns, with 50 and 70% methanol extracts forming a distinct cluster, while 100% methanol, lower methanol concentrations, and the seed sample diverged significantly. Interestingly, the seed sample, despite undergoing the same optimized extraction condition (50% methanol), exhibited substantially lower phenolic content (459.1 µg/g), with a divergent profile in both PCA and heatmap.

## 4. Discussion

It is well established that the TPC and TFC ratios in crops and natural edible sources are widely recognized for their beneficial impacts on human health [9,20,31]. Numerous studies have exhibited that these two factors are associated with various biological properties [4,5,9,19,22]. Previous research has also indicated that TPC and TFC are influenced by several extraction parameters, including solvent, time, pressure, temperature, and pH [3,5,9,15]. In particular, polar solvents have been extensively utilized due to their superior extraction efficiency [3,5,26]. Our findings clearly indicate that solvent polarity has a positive impact on the TPC pattern. Specifically, previous reports suggest that low polarity solvents, such as hexane, chloroform, and ethyl acetate, are less effective for extraction compared to high polarity solvents like methanol and water [3,15,22]. Methanol was prioritized as the extraction solvent to ensure exhaustive recovery of the target polar flavone glucosides (**11**–**13**), owing to its high dielectric constant. This choice provided a baseline of maximum extractability, serving as a reference for subsequent optimization with food-grade solvents such as ethanol. Interestingly, the acetonitrile and ethanol extracts exhibited significantly lower TPC and TFC ratios compared to those extracted with methanol and water (Figure 1A,B). Additionally, while previous studies indicated that acetone consistently shows greater efficacy in extracting phenolic and flavonoid compositions compared to water-based extraction methods [9], our findings demonstrated an opposite trend. This phenomenon may be attributed to the distinct physicochemical properties of individual phenolic and flavonoid components present in plant sources [20,22]. Supporting the above results, methanol and water emerged as efficient solvents for recovering TPC and TFC ratios from spinach leaves. The predominant antioxidant activities observed in methanol and water extracts were consistent with their higher TPC and TFC. In contrast, the extract obtained using acetonitrile, classified as a polar aprotic solvent, exhibited comparatively lower antioxidant abilities, correlating with its reduced TPC and TFC levels. This characteristic limits its effectiveness in solubilizing phenolics, which generally require both hydrogen bonding and polar interactions for optimal extraction [14,24]. These results correspond with previous findings indicating that aqueous mixtures of methanol or ethanol offer superior extraction efficiency for antioxidant-related phenolics and flavonoids due to their capacity to form strong hydrogen bonds and effectively interact with polar phenolic structures [3,22]. Consequently, we have confidence that the antioxidant capacities of methanol and water solvent extracts correlate directly with phenolic and flavonoid contents in spinach leaves [4,14,20,22]. Regarding DNA protection, the results suggest that phenolic compounds and other antioxidant-related metabolites present in spinach leaves may be key contributors to DNA protection [7,10,34]. For tyrosinase inhibition, the results showed that methanol and water extracts displayed potent inhibitory properties. This phenomenon may be attributed to differences in phenolic contents, as similarly reported in earlier studies on other crop sources [7,27,34]. While cell-based models offer greater physiological insight, this study focuses on natural product chemistry, specifically the isolation, structural elucidation, and biochemical validation of winter spinach phenolics. These enzymatic and DNA-level assays confirm the intrinsic bioactivity of the identified metabolites, providing a fundamental basis for future in vivo investigations. Notably, the enhanced ABTS radical scavenging activities of the 50% methanol extracts compared to DPPH suggest that these extracts likely utilize both chain-breaking and hydrogen-donating properties, whereas DPPH scavenging primarily reflects hydrogen-donating capacities [10,37]. Tyrosinase inhibitory activities may also be influenced by the levels and profiles of phenolic phytochemicals, specifically the hydroxyl groups and catecholic types of phenolics [9]. Our data demonstrate that the 50% methanol extract effectively prevents hydroxyl radical-induced DNA nicking, supporting its potential as an ROS-protective agent [10,13,27,34]. Overall, the 50% methanol extract could serve as a valuable ingredient for preventing metabolic disorders. In contrast to earlier reports on summer- or greenhouse-cultivated spinach [8,12,25,28,30], which primarily focused on qualitative profiling of glycosylated flavonoids using HPLC-DAD or LC-MS, the present study provides high-resolution UPLC-Q-TOF-MS/MS data combined with NMR-based structural confirmation specifically for the Korean cold-cultivated *Allseason* cultivar. The total content of flavone glucuronides (**11**–**13**) obtained under our optimized conditions (6525.6 μg/g) exceeds the corresponding values previously reported for non-winter spinach cultivars [8,25], supporting the hypothesis that low-temperature cultivation enhances flavonoid accumulation as a cold-stress response. Furthermore, while earlier studies typically reported one or two bioactivity measurements (most commonly DPPH or total antioxidant capacity), the present work integrates five complementary assays (DPPH, ABTS, FRAP, DNA protection, and tyrosinase inhibition), allowing both radical-scavenging and electron-donating mechanisms to be evaluated in parallel.

The analysis technique of LC coupled with mass spectrometry offers remarkable information regarding the demonstration of phenolic phytochemicals with molecular weight in natural sources, foods, and crops [5,28,31]. Moreover, this skill has proven to be a powerful analytical platform for the structural elucidation and compositional profiling of complex natural matrices [25,33]. According to the individual and total phenolic phytochemical patterns in winter spinach leaves, the current results exhibited considerable differences when compared with the previous studies [8,12,28,30,31]. These differences may be attributed to multiple contributing factors, including spinach cultivar/genotype, harvest stage, cultivation season, light intensity, temperature, and soil conditions, in addition to extraction parameters. Bergquist et al. [9] reported that flavonoid content in baby spinach changes significantly with plant age and storage, while Watanabe and Ayugase [12] demonstrated that low-temperature cultivation enhances flavonoid accumulation in winter spinach. Dzakovich et al. [30] further showed that spinach flavonoid profiles vary substantially among genetically diverse cultivars. The present research was conducted to establish a reliable method for the identification of phenolic phytochemicals in spinach leaves through UPLC-Q-TOF-MS/MS analysis. While colorimetric assays provided a preliminary screening of the total phenolic load, the definitive quantification was performed via validated UPLC-Q-TOF-MS/MS and NMR. This dual-analytical approach overcomes the selectivity limitations of traditional methods by providing high-resolution, metabolite-specific data. Furthermore, the structural identification through NMR spectroscopic analysis provides a definitive confirmation of the major metabolites. The chemical structures of three isolated phenolics **11**–**13** were elucidated by NMR spectroscopy and identified as follows: spinatoside (**11**), jaceidin-4′-β-D-glucuronide (**12**), and 5,3′,4′-trihydroxy-3-methoxy-6,7-methylenedioxyflavone-4′-β-D-glucuronide (**13**). The observed chemical shifts and coupling constants in the ^1^H and ^13^C NMR spectroscopic data are consistent with the aglycone moieties and glycosidic linkages characteristic of these compounds. Comprehensive NMR data, as presented in the supplementary information (Figures S13–S20), support the assignments made using UPLC-Q-TOF-MS/MS and reinforce the structural accuracy of the identified derivatives.

The development of functional foods and nutraceuticals has increasingly focused on phytochemicals, which exhibit a wide range of pharmaceutical properties [4,8,10,11,17,18,27]. Based on the above reasons, numerous studies have aimed to optimize extraction conditions to efficiently recover bioactive compounds [4,9,12,14,15,20,21,26]. Among various parameters, the choice of solvent is considered the most critical factor [3,7,9,22]. In our research, the absence of detectable phenolics in hexane, chloroform, and other low-polarity solvents is likely due to the sugar moieties in the chemical structures of the phenolics, which caused polarity incompatibility. These findings are consistent with previous reports indicating that low polarity solvents, such as chloroform and ethyl acetate, are less effective for extracting phenolics compared to high polarity solvents like methanol and water [3,7,26]. This emphasizes the importance of selecting appropriate extraction conditions, as studies have successfully increased phenolic yields through systematic optimization. Methanol demonstrated the highest extraction efficiency for phenolics from spinach leaves. While the extraction efficiency of phenolics from vegetables, fruits, and crops is generally influenced by maceration temperature [23], our results indicated that the main phenolic contents in winter spinach leaves were not significantly affected by temperature variations, with a slightly higher yield observed at 35 °C. Moreover, the combination of polar solvents with water has proven effective for maximizing phenolic recovery [7,20,22,26]. The reduction in yields at higher methanol concentrations may be due to insufficient water content to disrupt cellular matrices and release bound polar compounds, particularly glycosylated phenolics [3,5,7,26]. These comparative analyses highlight the importance of optimizing extraction conditions. In the present work, extraction optimization was carried out using a one-factor-at-a-time design, varying solvent type, methanol–water ratio, extraction time, and temperature sequentially. The condition selected (50% methanol, 35 °C, 72 h) represents the best among those tested. Statistical designs such as response surface methodology (RSM) based on Box–Behnken or central composite designs have been increasingly applied to phenolic extraction from various plant materials, including spinach by-products [38] and other leafy medicinal plants [39], and will be adopted in our follow-up work using food-grade solvents. Regarding solvent selection, the screening showed that phenolics **11**–**13** were extracted effectively only by methanol: hexane, chloroform, ethyl acetate, acetone, and acetonitrile yielded no detectable amounts; water recovered only 218.3 μg/g; and ethanol yielded only 20.3 μg/g of phenolic **11**, with **12** and **13** in trace amounts (Figure 3A; Table 2). Methanol was therefore used as the analytical reference solvent to establish the maximum extractable baseline of phenolics **11**–**13** [26]. Building on this baseline, further research is needed to investigate safer alternatives such as aqueous ethanol, natural deep eutectic solvents (NaDES), and subcritical water for establishing a food-grade extraction system suitable for nutraceutical applications [40]. This research is the first to demonstrate that using 50% methanol for 72 h at 35 °C yields the highest efficiency in phenolic extraction from spinach leaves. Despite extensive research on spinach, our study offers distinct originality as the first to systematically quantify three major flavone glucuronides (**11**–**13**) under optimized extraction parameters with NMR confirmed structures. Unlike prior generic profiling, our high-resolution data on the influence of extraction variables provide a practical framework for the standardized production of spinach-derived functional materials.

The evaluation of isolated phenolics **11**–**13** revealed significant biofunctional potential. Although the radical scavenging capacities of these three phenolics were lower than those of positive controls, the presence of various phenolic constituents, including patuletin, spinacetin, jaceidin, and methylene dioxyflavone derivatives in the spinach leaves, supports their potential as valuable natural antioxidants for the development of functional and health-promoting food products [8,26,31]. It is commonly observed that antioxidant abilities measured by DPPH and ABTS assays yield comparable responses, attributed to their radical scavenging mechanisms involving both hydrogen atom transfer and single electron transfer [27]. In contrast, the FRAP and DNA protection assays show a strong correlation, reflecting their shared dependence on electron donating capacity [17]. The observed phenomena between the two assay groups are likely due to differences in their underlying reaction principles and the distinct physicochemical properties of the phenolics [13].

The multivariate statistical approach, including PCA and hierarchical clustering, provides significant visualization of similarities and differences in extraction efficiency. In the solvent-based extraction analysis, the dominance of PC1 (over 99%) limits the interpretative value of the PCA for mechanistic insights; therefore, it is recommended to consider adding more variables for a more comprehensive analysis in future studies. The failure of solvents such as hexane, chloroform, ethyl acetate, and acetonitrile to recover phenolics demonstrates the limited solubility of these polar phenolics in non-polar or aprotic environments. These trends underscore the critical influence of solvent polarity and hydrogen bonding capacity on phenolic extraction [7,24,26]. Interestingly, extraction with 100% methanol yielded lower values than 50–70%, suggesting that the presence of water facilitates phenolic diffusion by enhancing matrix permeability, tissue swelling, and mass transfer [7,15,24]. This synergistic role of water is particularly important for extracting glycosylated or glucuronidated phenolics, which often reside within vacuoles or cell walls [26]. This finding strongly supports the notion of tissue-specific localization of these phenolics, which appear to accumulate predominantly in leafy tissues rather than in seeds. Collectively, these data confirm that both solvent polarity and methanol concentration critically modulate the extractability of phenolic derivatives in spinach leaves. To enhance the interpretative value of PCA and mechanistic insights, future studies should consider incorporating additional variables. The multivariate analysis provides not only a visualization of extraction trends but also mechanistic insight into the solvent–phenolic interactions.

## 5. Conclusions

The present research conducted a comparative evaluation of bioactive factors (TPC and TFC), antioxidant potential (DPPH, ABTS, FRAP, and DNA protection), and tyrosinase inhibitory properties in various solvent extracts of spinach leaves. The extract was subjected to fractionation and identification using silica gel column chromatography, MPLC, NMR spectroscopy, and UPLC-Q-TOF-MS/MS analysis. The beneficial factors and biological activities varied significantly depending on the solvent system, specifically, 50% methanol extract exhibited the most predominant ratios compared to other solvents. Twelve phenolics were elucidated, including patuletin (**1**, **2**, **5**, and **7**), spinacetin (**3**, **6**, **9**, and **10**), spinatoside (**11**), jaceidin (**12**), and methylenedioxyflavone-glucuronide (**13** and **14**) derivatives. Among them, phenolics **11**–**13** were the predominant components, with optimal extraction achieved using 50% methanol at 35 °C for 72 h, ranked as follows: **11** (3366.2 μg/g) > **13** (1626.9 μg/g) > **12** (1532.5 μg/g). PCA and hierarchical clustering revealed distinct phenolic patterns in different extraction systems. Additionally, phenolics **11** and **12** exhibited strong antioxidant activities against radicals with IC_50_ values of 83.7 and 57.6 μM in the DPPH assay and 29.5 and 21.9 μM in the ABTS method, outperforming other biofunctional properties. However, 50% methanol is unsuitable for direct food applications, none of the food-compatible solvents tested achieved comparable extraction efficiency, supporting its role here as an analytical reference. Modified food-grade protocols (e.g., aqueous ethanol-based systems) will be developed in subsequent work. Although the biological activities reported here were evaluated only through in vitro assays and require further validation by in vivo studies, these findings indicate that spinach leaves may be utilized as a promising natural resource for developing functional food ingredients. Further research is also needed to assess the applicability of these optimized extraction conditions to other spinach varieties.

## Figures and Tables

**Table 1:** List of identified phenolic derivatives in leaves of winter spinach by UPLC-Q-TOF-MS/MS analysis.

Peak	RT (min)	Elemental Composition	Calculated Mass [M] (*m*/*z*)	Calculated Mass [M-H]^−^ (*m*/*z*)	Observed Ions [M-H]^−^ (*m*/*z*)	Mass Error (ppm)	Fragment Ions [M-H]^−^ (*m*/*z*)	Identification	Reference	Supplementary Figure
1	4.02	C_33_H_40_O_22_	788.2011	787.1938	787.1911	0.258	315.0126 331.0420	patuletin-3-*O*-β-D-glucopyranosyl-(1→6)-[β-D-apiofuranosyl-(1→2)]-β-D-glucopyranoside	Barkat et al., 2018 [8]; Koh et al., 2012 [25]; Watanabe & Ayugase, 2014 [12]	Figure S1
2	4.29	C_28_H_32_O_18_	656.1588	655.1515	655.1485	−0.545	315.0126 331.0443	patuletin-3-*O*-β-D-glucopyranosyl-(1→6)-β-D-glucopyranoside	Dzakovich et al., 2025 [28]; Kamiloglu, 2020 [30]; Watanabe & Ayugase, 2014 [12]	Figure S2
3	4.38	C_34_H_42_O_22_	802.2167	801.2095	801.2102	0.253	329.0280 345.0594	spinacetin-3-*O*-β-D-glucopyranosyl-(1→6)-[apiofuranosyl-(1→2)]-β-D-glucopyranoside	Dzakovich et al., 2025 [28]; Son et al., 2024 [29]; Watanabe & Ayugase, 2014 [12]	Figure S3
4	4.49	C_34_H_42_O_22_	802.2167	801.2095	801.2066	−1.523	329.0365 345.0564 467.1600	isomer of peak 3	-	-
5	4.64	C_42_H_46_O_24_	934.2379	933.2306	933.2295	−0.616	331.0420 787.1946	patuletin-3-*O*-β-D-(2″-β-coumaroylglucopyranosyl)-(1→6)-[β-D-apiofuranosyl-(1→2)]-β-D-glucopyranoside	Barkat et al., 2018 [8]; Cho et al., 2008 [31]; Koh et al., 2012 [25]	Figure S4
6	4.79	C_29_H_34_O_18_	670.1745	669.1672	669.1666	−0.519	329.0280 345.0594	spinacetin-3-*O*-β-D-glucopyranosyl-(1→6)-β-D-glucopyranoside	Barkat et al., 2018 [8]; Cho et al., 2008 [31]; Kamiloglu, 2020 [30]	Figure S5
7	4.85	C_43_H_48_O_25_	964.2484	963.2406	963.2368	−0.746	177.1600 331.0365 787.1946	patuletin 3-*O*-(2″-feruloylglucosyl)-(1→6)-[apiofuranosyl-(1→2)]-glucopyranoside	Cho et al., 2008 [31]; Barkat et al., 2018 [8]; Koh et al., 2012 [25]; Singh et al., 2017 [35]	Figure S6
8	4.87	C_43_H_48_O_25_	964.2484	963.2406	963.2310	−1.445	331.0432 787.1943	isomer of peak 7	-	-
9	4.94	C_43_H_48_O_24_	948.2535	947.2462	947.2464	0.710	119.0503 345.0594 801.2066	spinacetin-3-*O*-β-D-(2″-β-coumaroylglucopyranosyl)-(1→6)-[β-D-apiofuranosyl-(1→2)]-β-D-glucopyranoside	Barkat et al., 2018 [8]; Cho et al., 2008 [31]; Koh et al., 2012 [25]	Figure S7
10	5.07	C_44_H_50_O_25_	978.2641	977.2568	977.2584	0.857	345.0517 801.2102	spinacetin-3-*O*-β-D-(2″feruloylglucopyranosyl) (1→6)-[β-D-apiofuranosyl(1→2)]-β-D-glucopyranoside	Cho et al., 2008 [31]; Dzakovich et al., 2025 [28]; Koh et al., 2012 [25]; Watanabe & Ayugase, 2014 [12]	Figure S8
11	5.74	C_23_H_22_O_14_	522.1009	521.0936	521.0939	−0.649	315.0126 330.0365 345.0617	spinatoside	Dzakovich et al., 2025 [28]; Kamiloglu, 2020 [30]; Koh et al., 2012 [25]; Watanabe & Ayugase, 2014 [12]	Figures S9 and S13–S16
12	6.10	C_24_H_24_O_14_	536.1166	535.1093	535.1080	−0.128	329.0280 344.0517 359.0765	jaceidin-4′-β-D-glucuronide	Dzakovich et al., 2025 [28]; Kamiloglu, 2020 [30]; Koh et al., 2012 [25]; Watanabe & Ayugase, 2014 [12]	Figures S10, S17 and S18
13	6.76	C_23_H_20_O_14_	520.0853	519.0780	519.0777	0.694	328.0211 343.0455	5,3′,4′-trihydroxy-3-methoxy-6,7-methylenedioxyflavone-4′-β-D-glucuronide	Kamiloglu, 2020 [30]; Koh et al., 2012 [25]; Watanabe & Ayugase, 2014 [12]	Figures S11, S19 and S20
14	7.12	C_24_H_22_O_14_	534.1009	533.0936	533.0942	0.638	299.0189 341.0360 357.0604	5,4′-dihydroxy-3,3′-dimethoxy-6,7-methylenedioxyflavone-4′-β-D-glucuronide	Kamiloglu, 2020 [30]; Koh et al., 2012 [25]; Watanabe & Ayugase, 2014 [12]	Figure S12

**Table 2:** Quantification of three major phenolic derivatives (**11–13**) in leaves and seeds of winter spinach under different extraction conditions.

Phenolics (µg/g) ^1^	Extraction Solvent for 24 h at 25 °C							
	Hexane	Chloroform	Ethyl Acetate	Acetone	Ethanol	Acetonitrile	Methanol	Water
11	ND ^2^	ND	ND	ND	20.3 ± 0.4 c	ND	1352.5 ± 46.4 a	58.1 ± 0.5 b
12	ND	ND	ND	ND	tr ^3^	ND	653.2 ± 19.2 a	6.0 ± 0.2 b
13	ND	ND	ND	ND	tr	ND	710.5 ± 14.8 a	154.2 ± 1.8 b
Total	-	-	-	-	20.3	-	2716.2	218.3
	% methanol in water for 72 h at 35 °C						Seeds (50% methanol for 72 h at 35 °C)	
	10	30	50	70	90	100		
11	1130.3 ± 28.5 d	2170.2 ± 61.1 b	3366.2 ± 120.4 a	3237.1 ± 68.6 a	2181.4 ± 70.3 b	1427.0 ± 36.9 c	118.3 ± 1.5 e	
12	674.7 ± 14.4 c	1026.4 ± 24.3 b	1532.5 ± 57.0 a	1488.6 ± 43.6 a	985.3 ± 22.4 b	697.6 ± 13.6 c	130.6 ± 3.3 d	
13	709.3 ± 13.6 e	1111.8 ± 30.1 d	1626.9 ± 32.6 a	1535.9 ± 42.0 b	1118.5 ± 29.1 c	754.4 ±16.6 f	210.2 ± 7.9 g	
Total	2514.3	4308.4	6525.6	6261.5	4285.2	2879.0	459.1	

^1^ All values are presented as the mean ± SD of pentaplicate determinations, content expressed as µg of each phenolic equivalents per g of dry weight. Means with different letters within a column for each sample indicate statistically significant difference (*p* < 0.05). ^2^ ND, not detected. ^3^ tr, trace < 0.001.

## Data Availability

The original contributions presented in this study are included in the article/Supplementary Materials. Further inquiries can be directed to the corresponding authors.

## Supplementary Materials

The following supporting information can be downloaded at: https://www.mdpi.com/article/10.3390/antiox15060686/s1, Table S1. Summary of phenolic compounds identified in winter spinach (*Spinacia oleracea* L.) leaves, including retention time, proposed identification, and the corresponding supplementary figures; Figure S1: MS/MS spectrum of phenolic phytochemical 1 using negative ion mode of UPLC-Q-TOF-MS and UPLC-Q-TOF-MS/MS analysis; Figure S2: MS/MS spectrum of phenolic phytochemical 2 using negative ion mode of UPLC-Q-TOF-MS and UPLC-Q-TOF-MS/MS analysis; Figure S3: MS/MS spectrum of phenolic phytochemical 3 using negative ion mode of UPLC-Q-TOF-MS and UPLC-Q-TOF-MS/MS analysis; Figure S4: MS/MS spectrum of phenolic phytochemical 5 using negative ion mode of UPLC-Q-TOF-MS and UPLC-Q-TOF-MS/MS analysis; Figure S5: MS/MS spectrum of phenolic phytochemical 6 using negative ion mode of UPLC-Q-TOF-MS and UPLC-Q-TOF-MS/MS analysis; Figure S6: MS/MS spectrum of phenolic phytochemical 7 using negative ion mode of UPLC-Q-TOF-MS and UPLC-Q-TOF-MS/MS analysis; Figure S7: MS/MS spectrum of phenolic phytochemical 9 using negative ion mode of UPLC-Q-TOF-MS and UPLC-Q-TOF-MS/MS analysis.; Figure S8: MS/MS spectrum of phenolic phytochemical 10 using negative ion mode of UPLC-Q-TOF-MS and UPLC-Q-TOF-MS/MS analysis; Figure S9: MS/MS spectrum of phenolic phytochemical 11 using negative ion mode of UPLC-Q-TOF-MS and UPLC-Q-TOF-MS/MS analysis; Figure S10: MS/MS spectrum of phenolic phytochemical 12 using negative ion mode of UPLC-Q-TOF-MS and UPLC-Q-TOF-MS/MS analysis; Figure S11: MS/MS spectrum of phenolic phytochemical 13 using negative ion mode of UPLC-Q-TOF-MS and UPLC-Q-TOF-MS/MS analysis; Figure S12: MS/MS spectrum of phenolic phytochemical 14 using negative ion mode of UPLC-Q-TOF-MS and UPLC-Q-TOF-MS/MS analysis; Figure S13: **^1^**H-NMR spectrum of phenolic phytochemical **11** (500 MHz, CD_3_OD); Figure S14: ^13^C–NMR spectrum of phenolic phytochemical **11** (125 MHz, CD_3_OD); Figure S15: DEPT 90 and 135 NMR spectra of phenolic phytochemical **11** (125 MHz, CD_3_OD); Figure S16: ^1^H−^1^H COSY, HMBC, and HMQC NMR spectra of phenolic phytochemical **11** (125 MHz, CD_3_OD); Figure S17: **^1^**H-NMR spectrum of phenolic phytochemical **12** (500 MHz, CD_3_OD); Figure S18: ^13^C–NMR spectrum of phenolic phytochemical **12** (125 MHz, CD_3_OD); Figure S19: **^1^**H-NMR spectrum of phenolic phytochemical **13** (500 MHz, CD_3_OD); Figure S20: ^13^C–NMR spectrum of phenolic phytochemical **13** (125 MHz, CD_3_OD).

## Abbreviations

The following abbreviations are used in this manuscript:

ABTS	2,2′-azino-bis(3-ethylbenzthiazoline-6-sulphonic acid)
DPPH	2,2-diphenyl-1-picrylhydrazyl
NMR	Nuclear magnetic resonance
UPLC-ESI-Q-TOF-MS/MS	Ultra high performance liquid chromatography coupled with electrospray ionization quadrupole time-of-flight mass spectrometry
HPLC	High performance liquid chromatography
