# Supplementary material for: Characterization of Phenolic Profiles Using UPLC-Q-TOF-MS/MS and NMR in the Biofunctional Fraction of Korean Winter Spinach (Spinacia oleracea L.) Leaves: Evaluation of Major Phenolics and Their Bioactivities Under Optimized Extraction Conditions
Source: Antioxidants (Basel). 2026 May 29;15(6):686. doi: 10.3390/antiox15060686 (PMC13295262; doi:10.3390/antiox15060686)
Supplement: Supplementary file 1 [file antioxidants-15-00686-s001.zip › antioxidants-4311047-supplementary.pdf]

Characterization of phenolic profiles using UPLC-Q-TOF-MS/MS and NMR in the biofunctional fraction of Korean winter spinach (*Spinacia oleracea* L.) leaves: Evaluation of major phenolics and their bioactivities under optimized extraction conditions

Supporting Information

I. **(Figure S1-S12) Fragmentation patterns of 12 phenolic phytochemicals through negative ion mode of UPLC-Q-TOF-MS/MS**

II. **(Figure S13-S20) ^{1}H-NMR and ^{13}C-NMR data on the isolated three major phenolic phytochemicals (11-13)**

Table S1. Summary of phenolic compounds identified in winter spinach (*Spinacia oleracea* L.) leaves, including retention time, proposed identification, and the corresponding supplementary figures

Peak	RT (min)	Proposed Identification	MS/MS Figure	NMR Figure
1	4.02	Patuletin-3-O-β-D-glucopyranosyl-(1→6)-[β-D-apiofuranosyl] derivative	Figs. S1-1, S1-2	–
2	4.29	Patuletin-3-O-β-D-glucopyranosyl-(1→6)-β-D-glucopyranoside	Figs. S2-1, S2-2	–
3	4.38	Spinacetin-3-O-β-D-glucopyranosyl-(1→6)-[β-D-apiofuranosyl] derivative	Figs. S3-1, S3-2	–
4	4.49	Isomer of peak 3	–	–
5	4.64	Patuletin-3-O-β-D-(2″-β-coumaroylglucopyranosyl) derivative	Figs. S4-1, S4-2	–
6	4.79	Spinacetin-3-O-β-D-glucopyranosyl-(1→6)-β-D-glucopyranoside	Figs. S5-1, S5-2	–
7	4.85	Patuletin 3-O-(2″-feruloylglucosyl)-(1→6)-[β-D-apiofuranoside]	Figs. S6-1, S6-2	–
8	4.87	Isomer of peak 7	–	–
9	4.94	Spinacetin-3-O-β-D-(2″-β-coumaroylglucopyranosyl) derivative	Figs. S7-1, S7-2	–
10	5.07	Spinacetin-3-O-β-D-(2″-feruloylglucopyranosyl)-(1→6) derivative	Figs. S8-1, S8-2	–
11	5.74	Spinatoside	Figs. S9-1, S9-2	Figs. S13–S16
12	6.10	Jaceidin-4′-β-D-glucuronide	Figs. S10-1, S10-2	Figs. S17, S18
13	6.76	5,3′,4′-trihydroxy-3-methoxy-6,7-methylenedioxyflavone-4′-β-D-glucuronide	Figs. S11-1, S11-2	Figs. S19, S20
14	7.12	5,4′-dihydroxy-3,3′-dimethoxy-6,7-methylenedioxyflavone derivative	Figs. S12-1, S12-2	–

Compounds **11–13** were structurally confirmed by NMR spectroscopy in addition to UPLC-Q-TOF-MS/MS analysis. Compounds **4** and **8** were tentatively assigned as structural isomers based on identical molecular masses and similar fragment ion patterns; no separate supplementary spectra are provided. The remaining compounds were putatively identified based on accurate mass measurements, MS/MS fragmentation patterns, and comparison with previously reported spinach metabolite data.

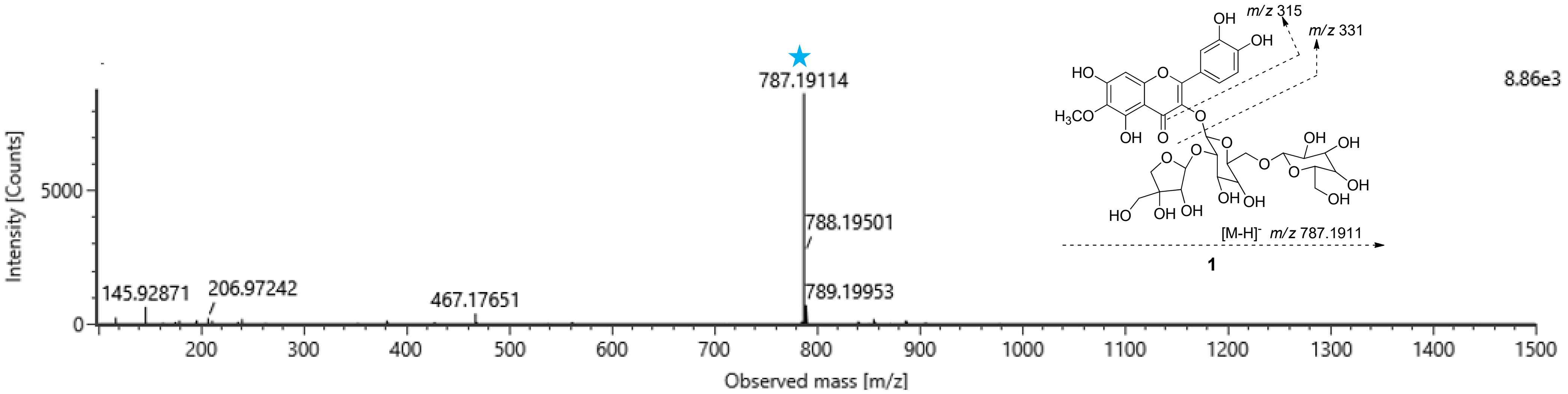

Figure S1-1. MS spectrum of phenolic phytochemical **1** using negative ion mode of UPLC-Q-TOF-MS analysis.

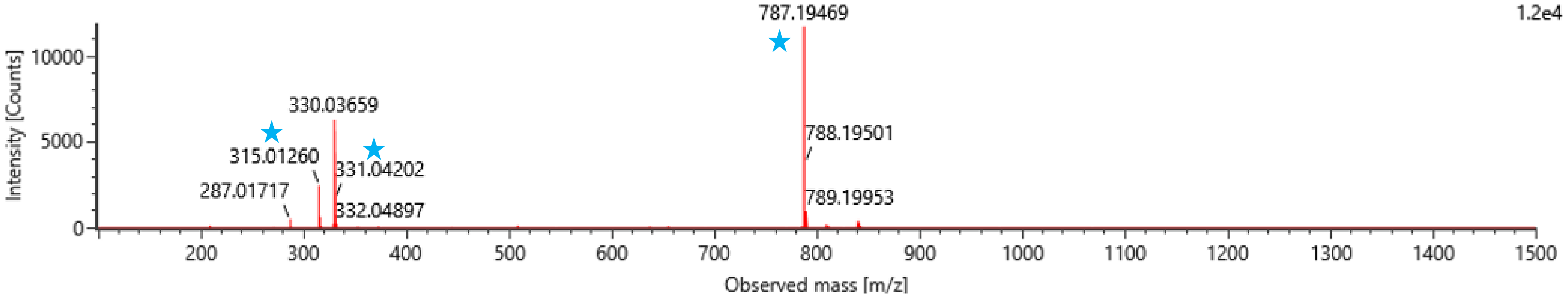

Figure S1-2. MS/MS spectrum of phenolic phytochemical **1** using negative ion mode of UPLC-Q-TOF-MS analysis.

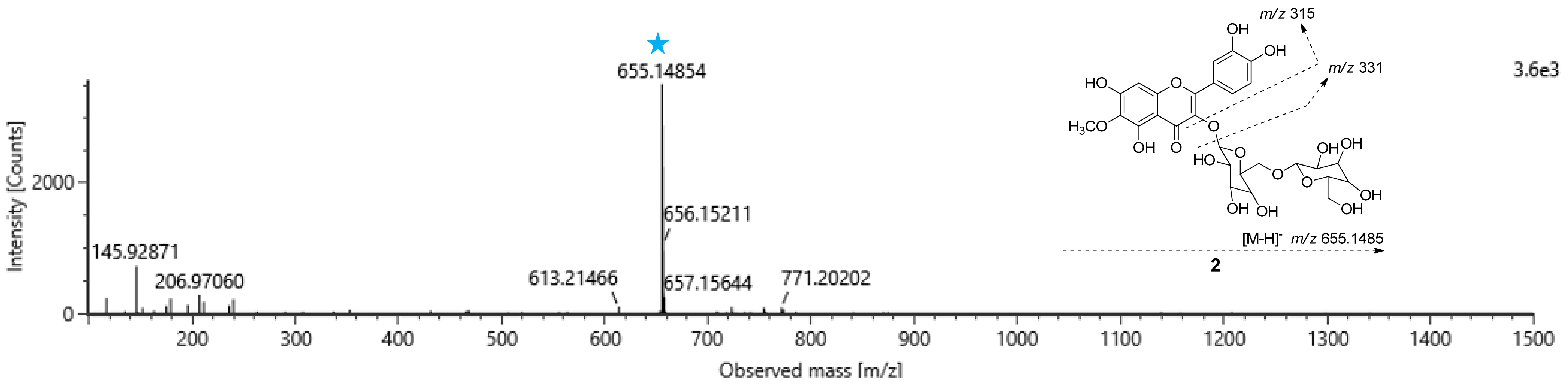

Figure S2-1. MS spectrum of phenolic phytochemical **2** using negative ion mode of UPLC-Q-TOF-MS analysis.

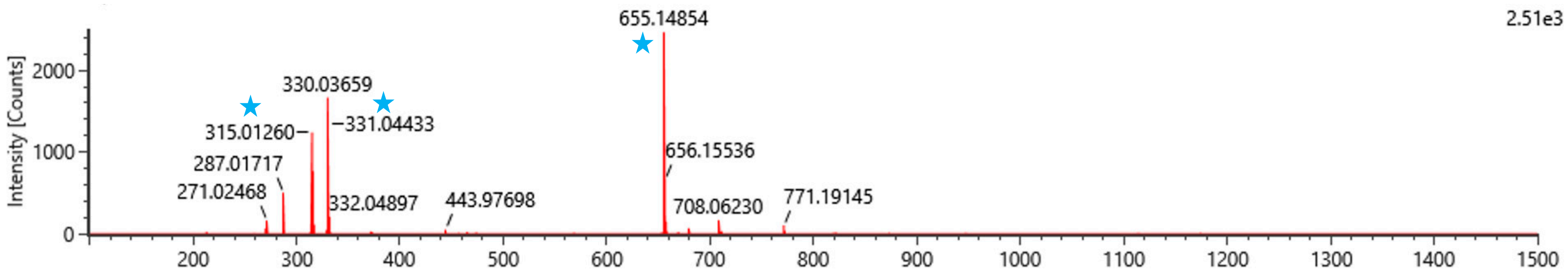

Figure S2-2. MS/MS spectrum of phenolic phytochemical **2** using negative ion mode of UPLC-Q-TOF-MS analysis .

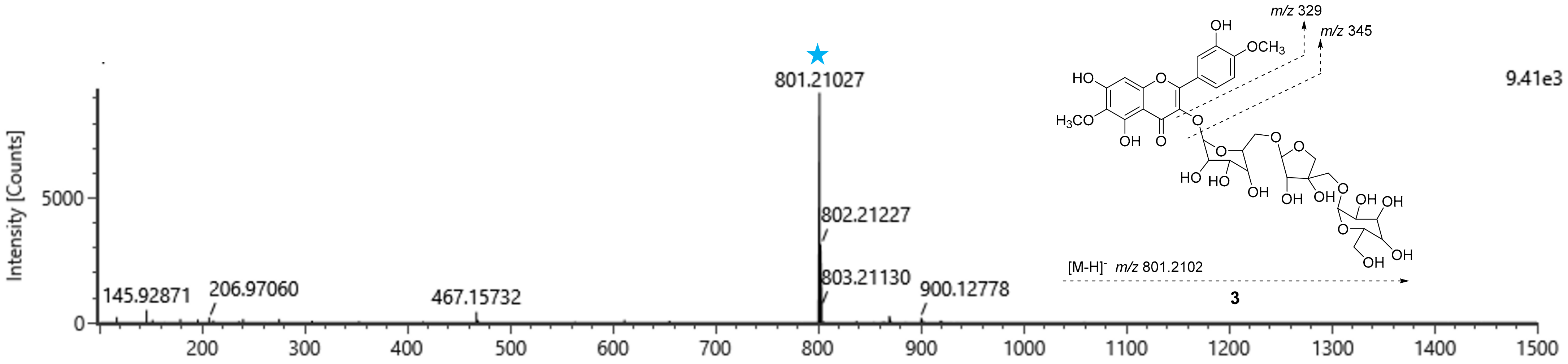

Figure S3-1. MS spectrum of phenolic phytochemical **3** using negative ion mode of UPLC-Q-TOF-MS analysis.

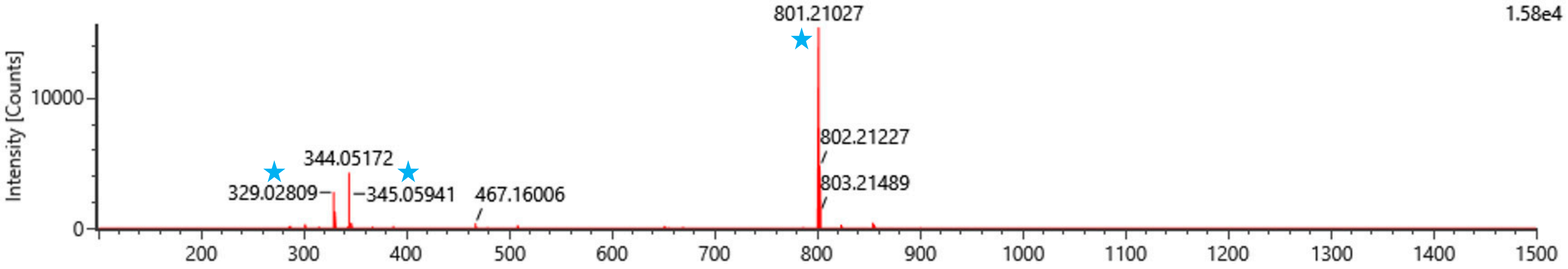

Figure S3-2. MS/MS spectrum of phenolic phytochemical **3** using negative ion mode of UPLC-Q-TOF-MS analysis.

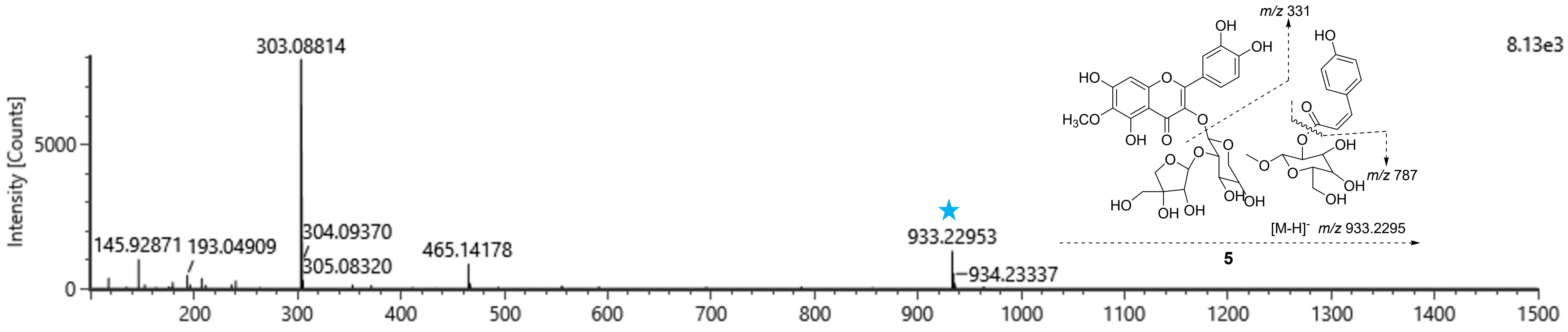

Figure S4-1. MS spectrum of phenolic phytochemical **5** using negative ion mode of UPLC-Q-TOF-MS analysis.

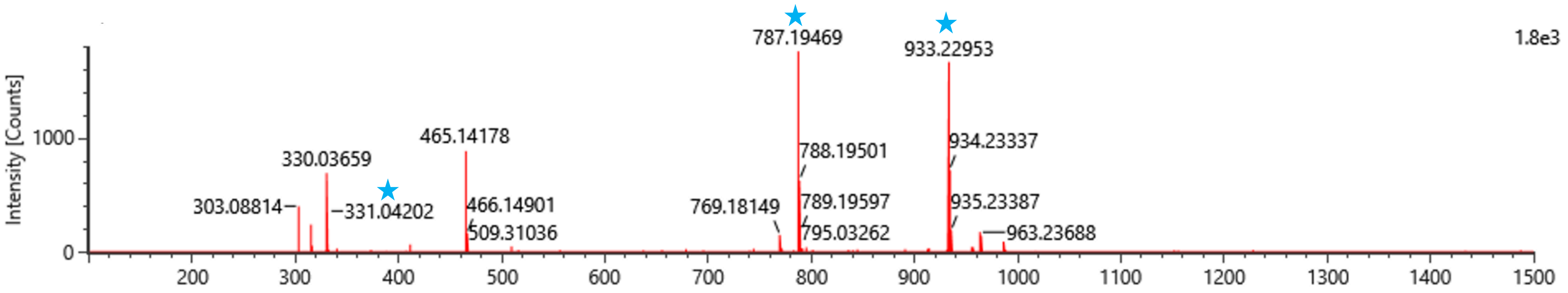

Figure S4-2. MS/MS spectrum of phenolic phytochemical **5** using negative ion mode of UPLC-Q-TOF-MS analysis.

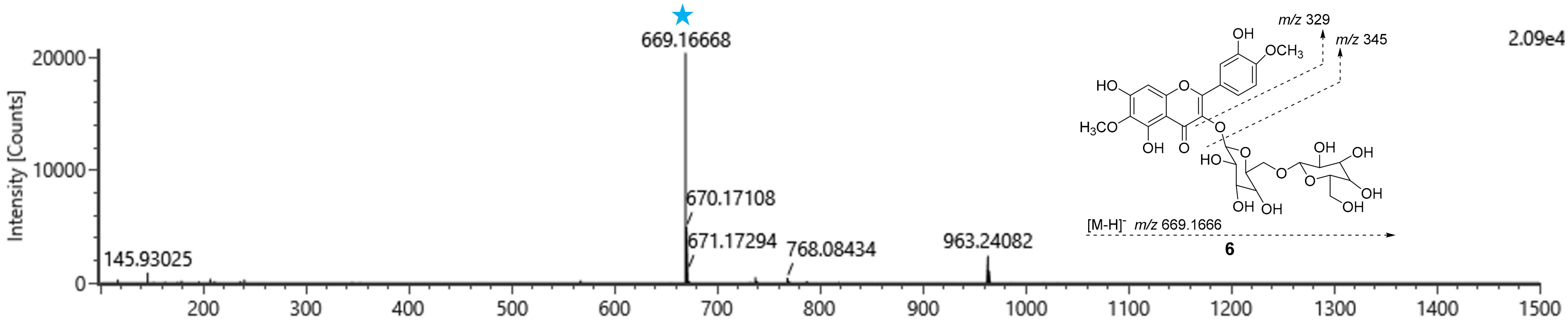

Figure S5-1. MS spectrum of phenolic phytochemical **6** using negative ion mode of UPLC-Q-TOF-MS analysis.

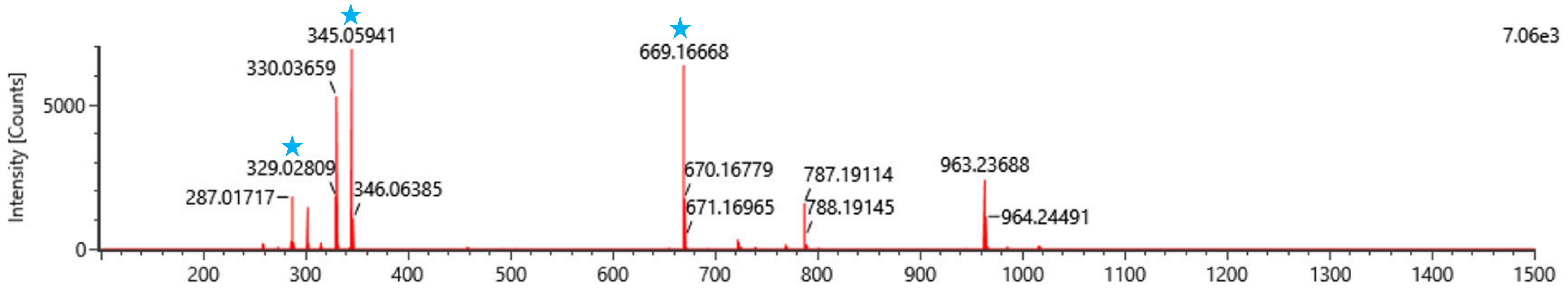

Figure S5-2. MS/MS spectrum of phenolic phytochemical **6** using negative ion mode of UPLC-Q-TOF-MS analysis.

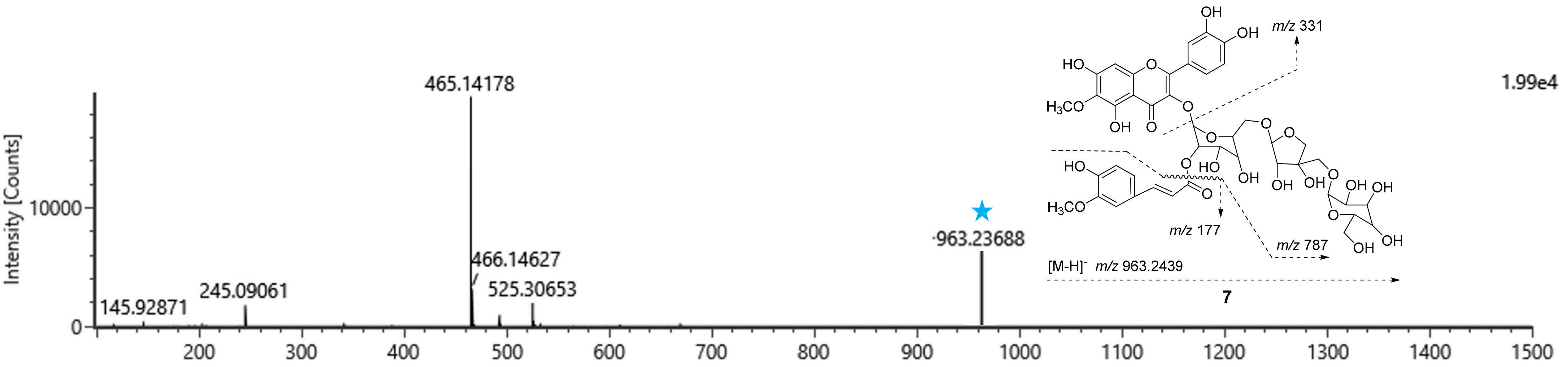

Figure S6-1. MS spectrum of phenolic phytochemical **7** using negative ion mode of UPLC-Q-TOF-MS analysis.

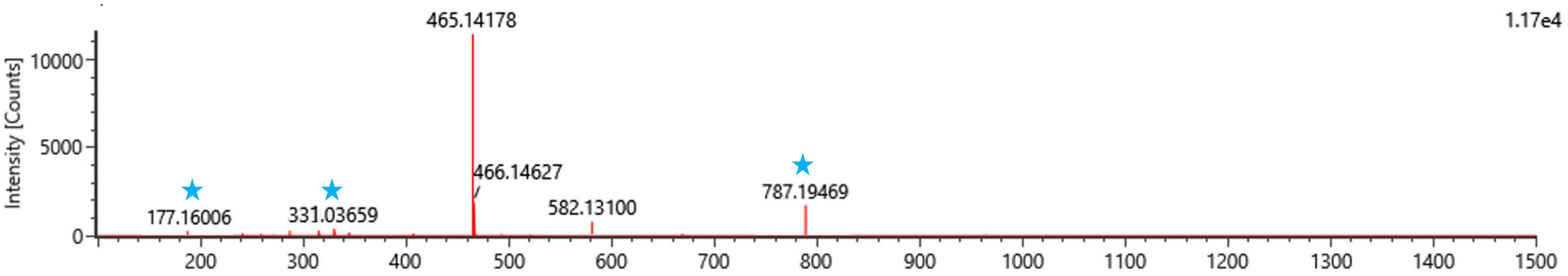

Figure S6-2. MS/MS spectrum of phenolic phytochemical **7** using negative ion mode of UPLC-Q-TOF-MS analysis.

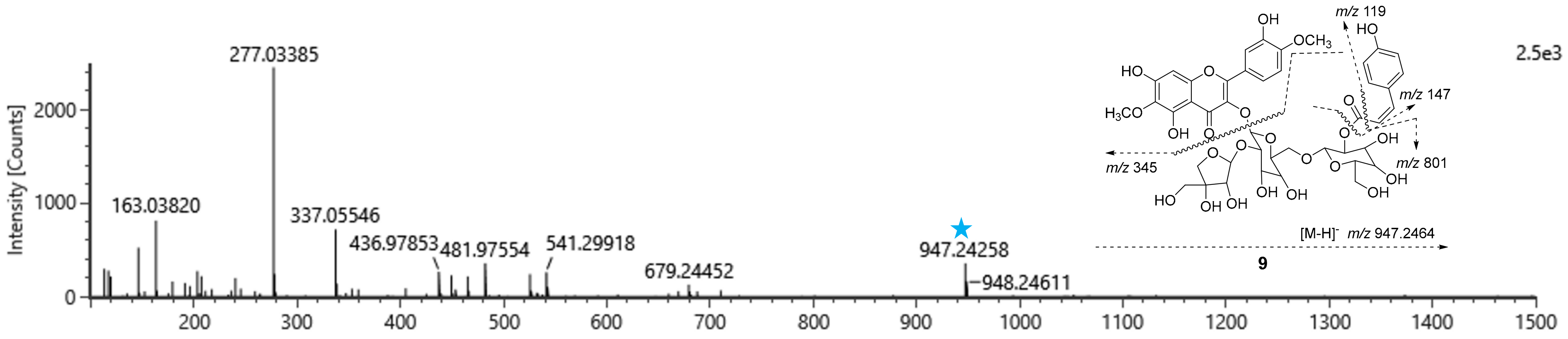

Figure S7-1. MS spectrum of phenolic phytochemical **9** using negative ion mode of UPLC-Q-TOF-MS analysis.

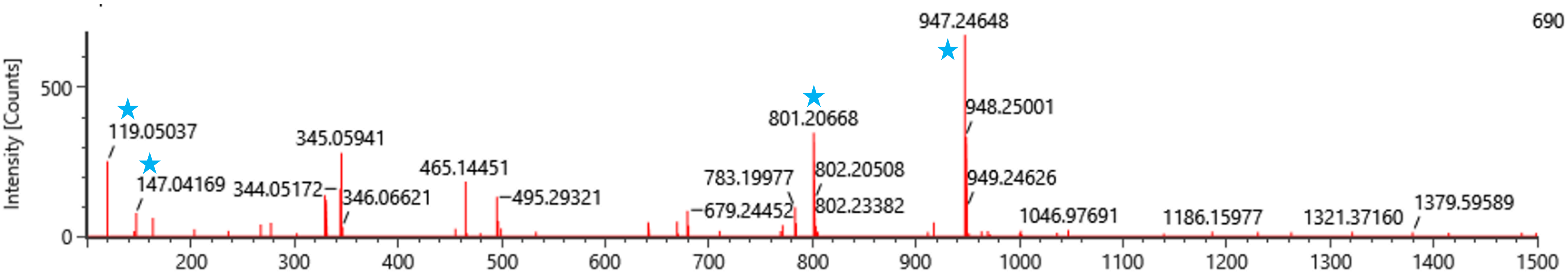

Figure S7-2. MS/MS spectrum of phenolic phytochemical **9** using negative ion mode of UPLC-Q-TOF-MS analysis.

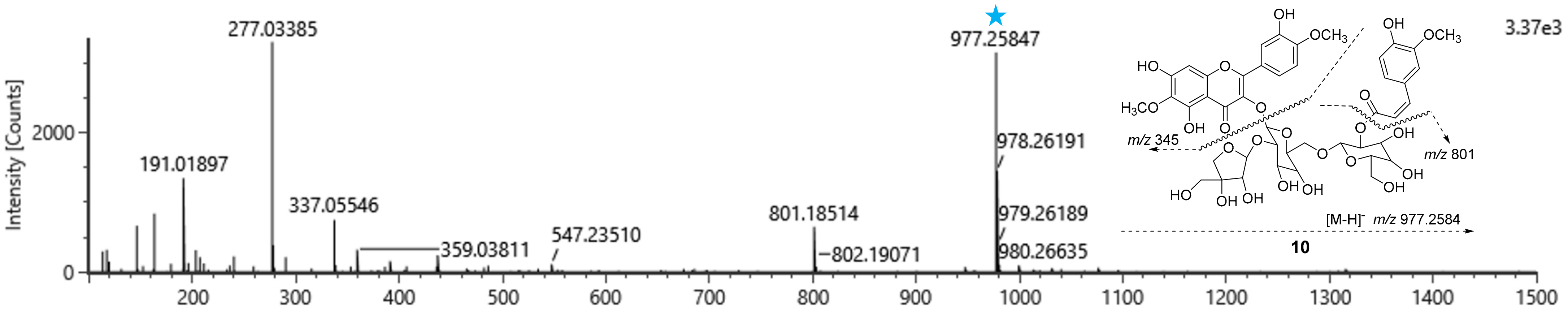

Figure S8-1. MS spectrum of phenolic phytochemical **10** using negative ion mode of UPLC-Q-TOF-MS analysis.

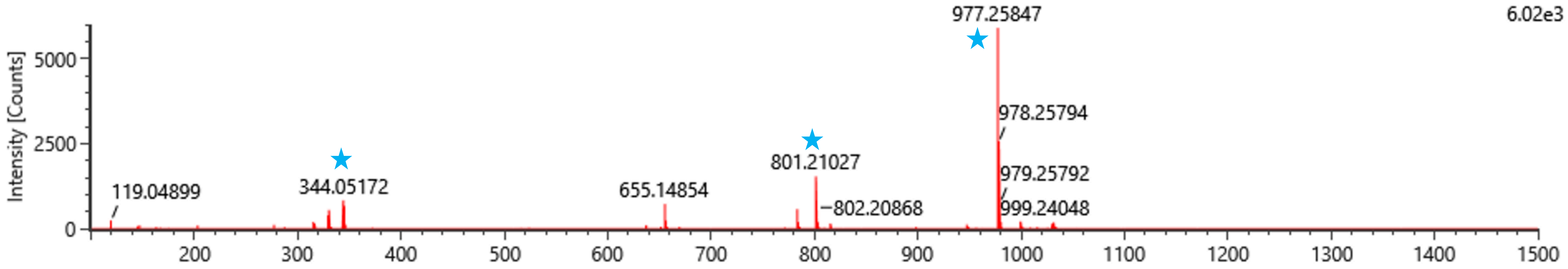

Figure S8-2. MS/MS spectrum of phenolic phytochemical **10** using negative ion mode of UPLC-Q-TOF-MS analysis.

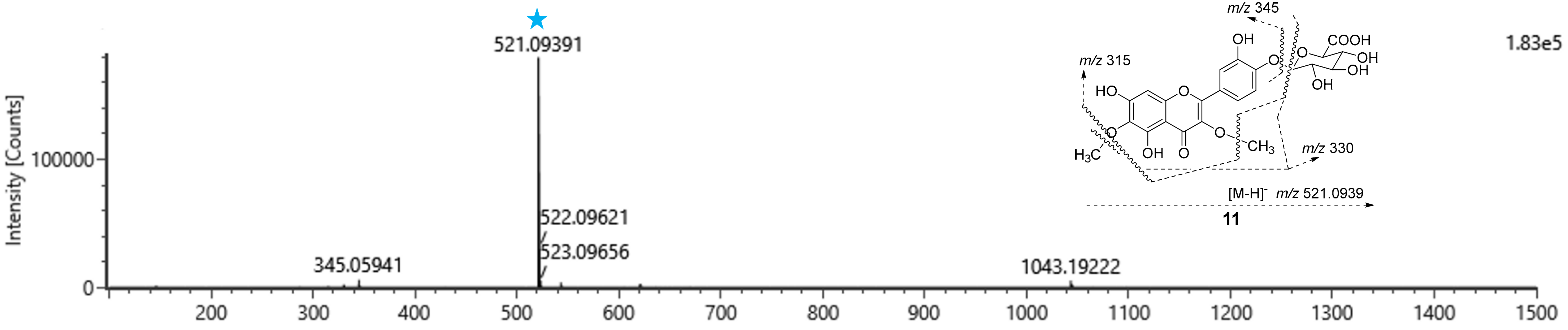

Figure S9-1. MS spectrum of phenolic phytochemical **11** using negative ion mode of UPLC-Q-TOF-MS analysis.

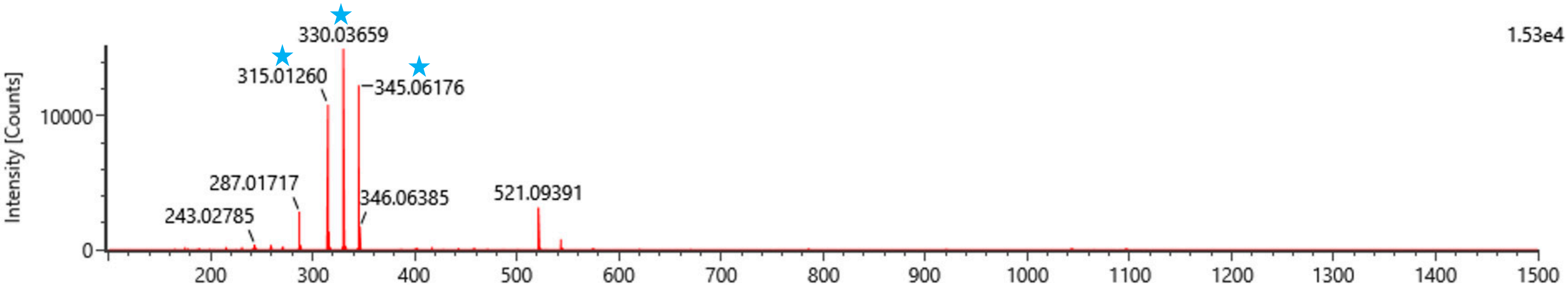

Figure S9-2. MS/MS spectrum of phenolic phytochemical **11** using negative ion mode of UPLC-Q-TOF-MS analysis.

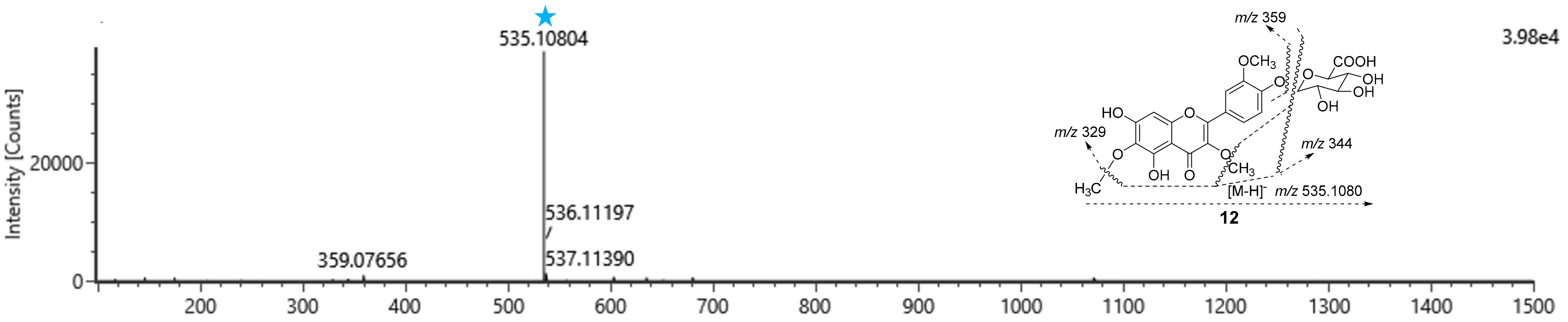

Figure S10-1. MS spectrum of phenolic phytochemical **12** using negative ion mode of UPLC-Q-TOF-MS analysis.

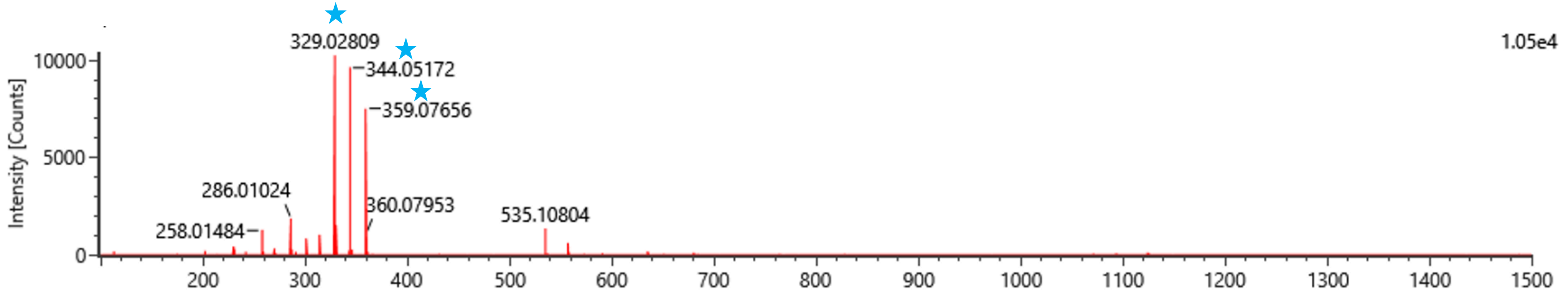

Figure S10-2. MS/MS spectrum of phenolic phytochemical **12** using negative ion mode of UPLC-Q-TOF-MS analysis.

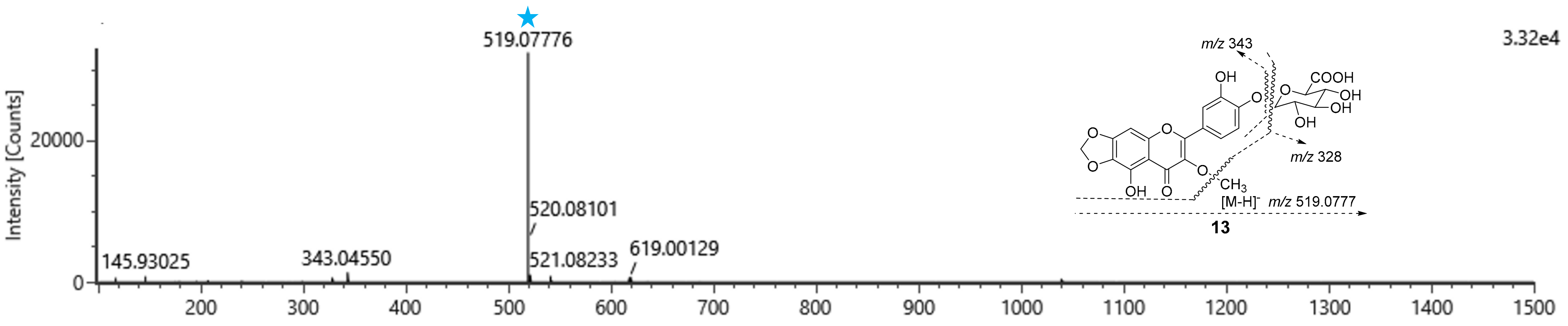

Figure S11-1. MS spectrum of phenolic phytochemical **13** using negative ion mode of UPLC-Q-TOF-MS analysis.

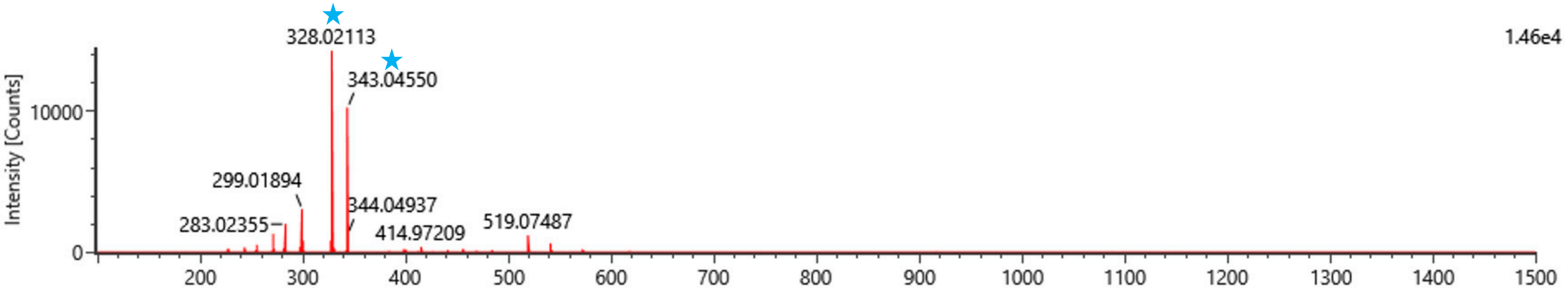

Figure S11-2. MS/MS spectrum of phenolic phytochemical **13** using negative ion mode of UPLC-Q-TOF-MS/MS analysis.

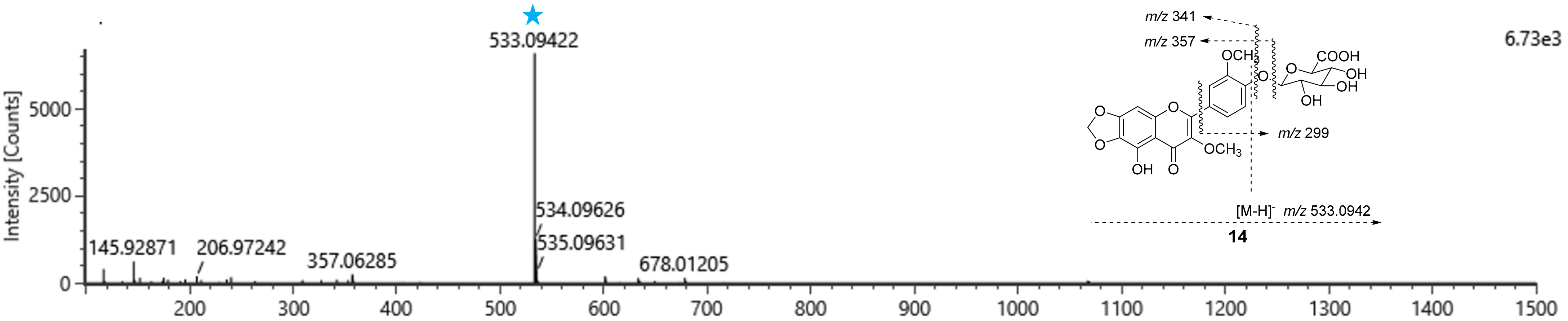

Figure S12-1. MS spectrum of phenolic phytochemical **14** using negative ion mode of UPLC-Q-TOF-MS analysis.

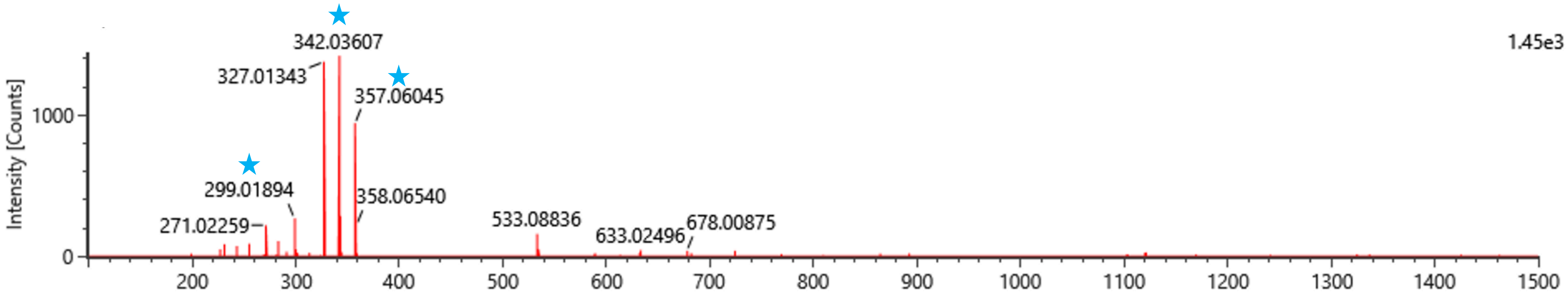

Figure S12-2. MS/MS spectrum of phenolic phytochemical **14** using negative ion mode of UPLC-Q-TOF-MS/MS analysis.

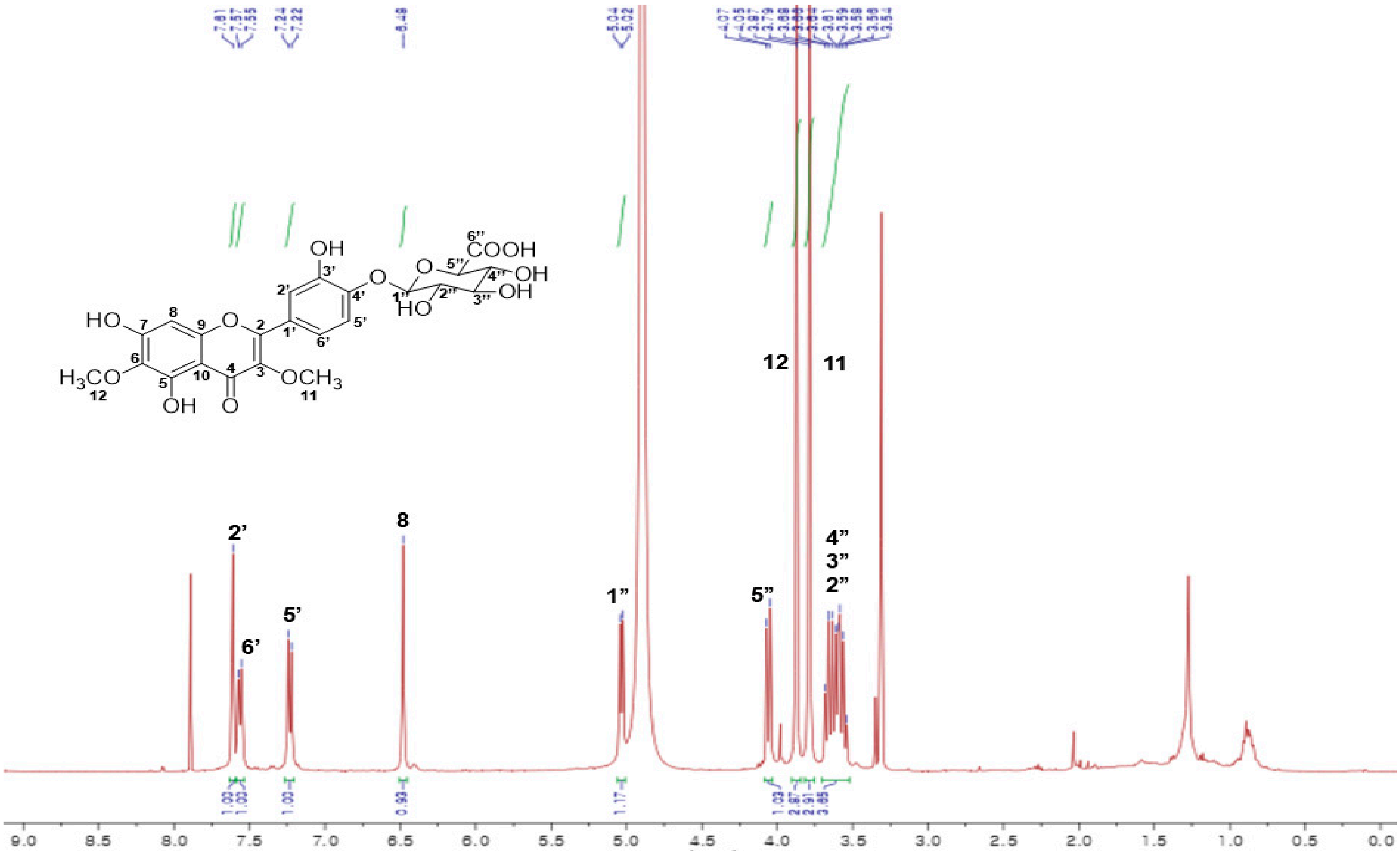

Figure S13. ^{1}H-NMR spectrum of phenolic phytochemical **11** (500 MHz, CD_3OD).

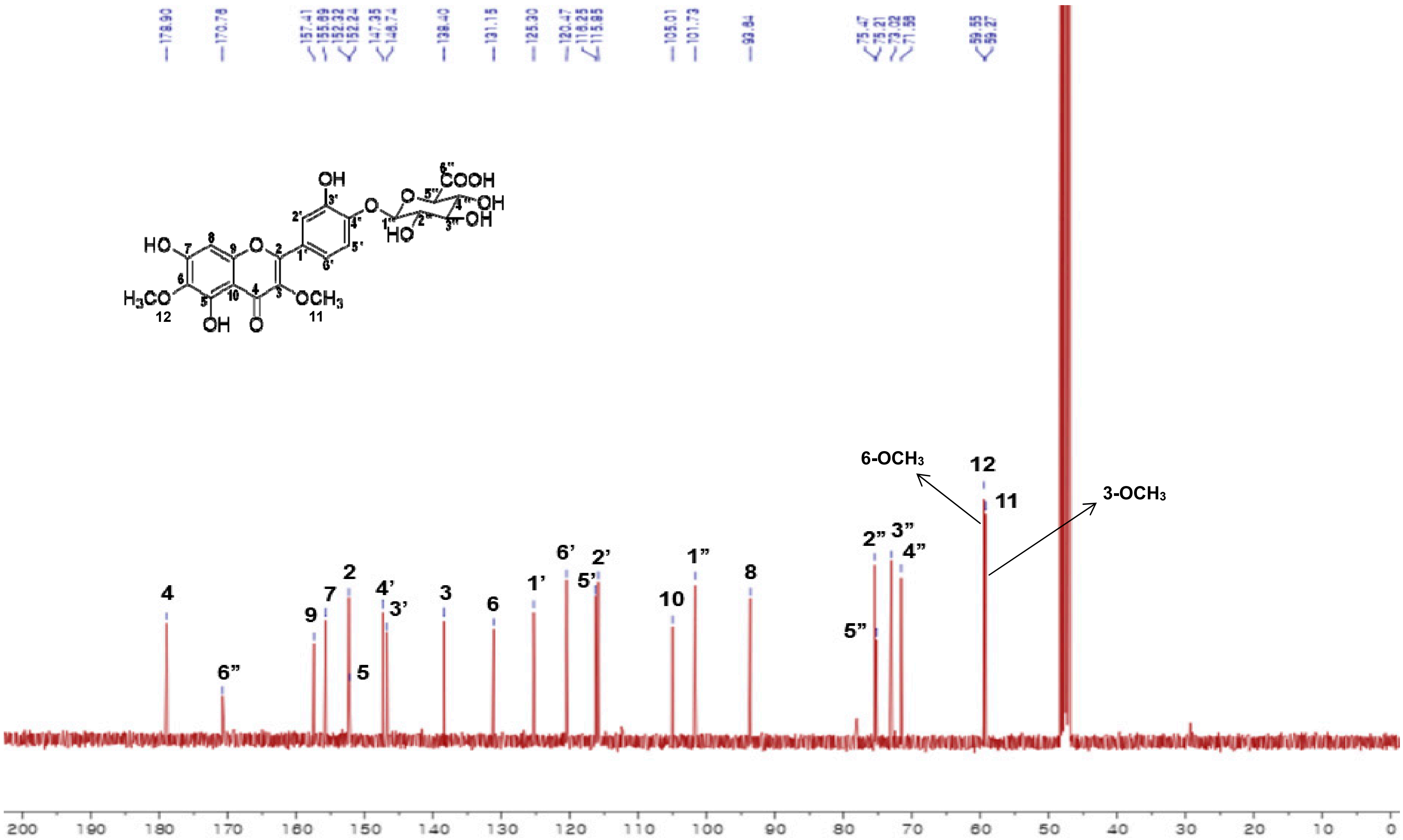

Figure S14. ^{13}C –NMR spectrum of phenolic phytochemical **11** (125 MHz, CD_3OD).

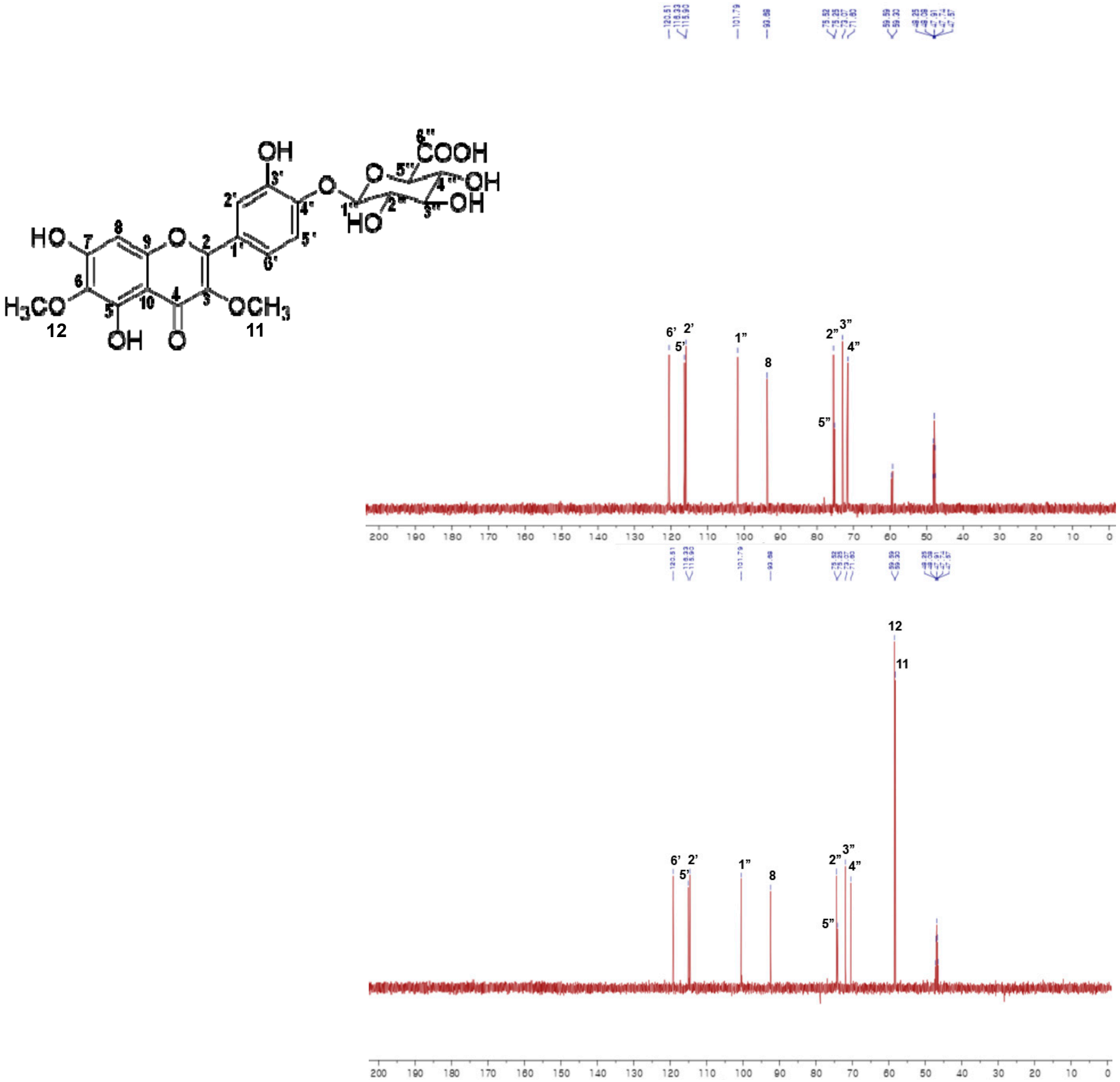

Figure S15. DEPT 90 and 135 NMR spectra of phenolic phytochemical **11** (125 MHz, CD_3OD).

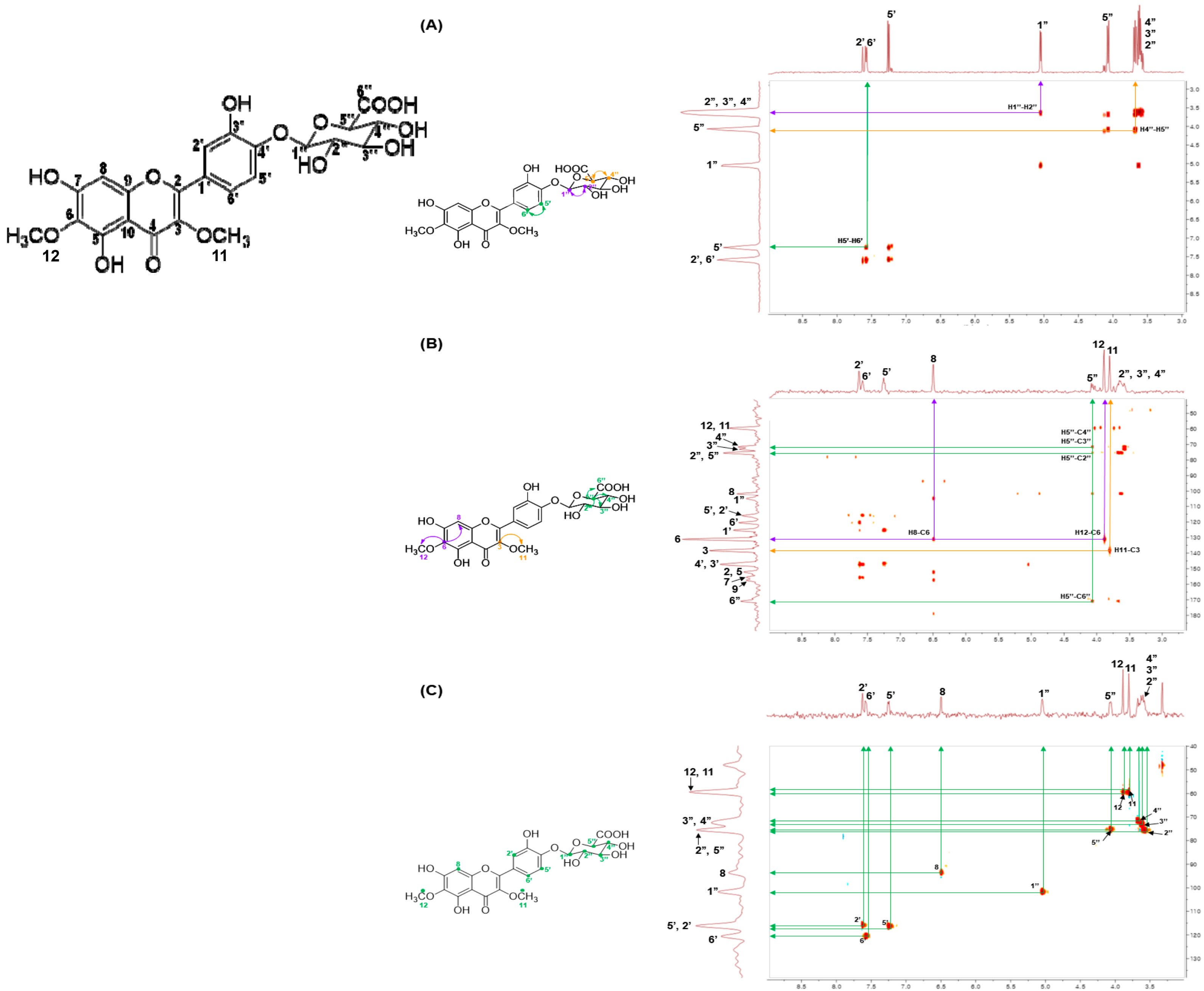

Figure S16. ^{1}H-^{1}H COSY, HMBC, and HMQC NMR spectra of phenolic phytochemical **11** (125 MHz, CD_3OD).

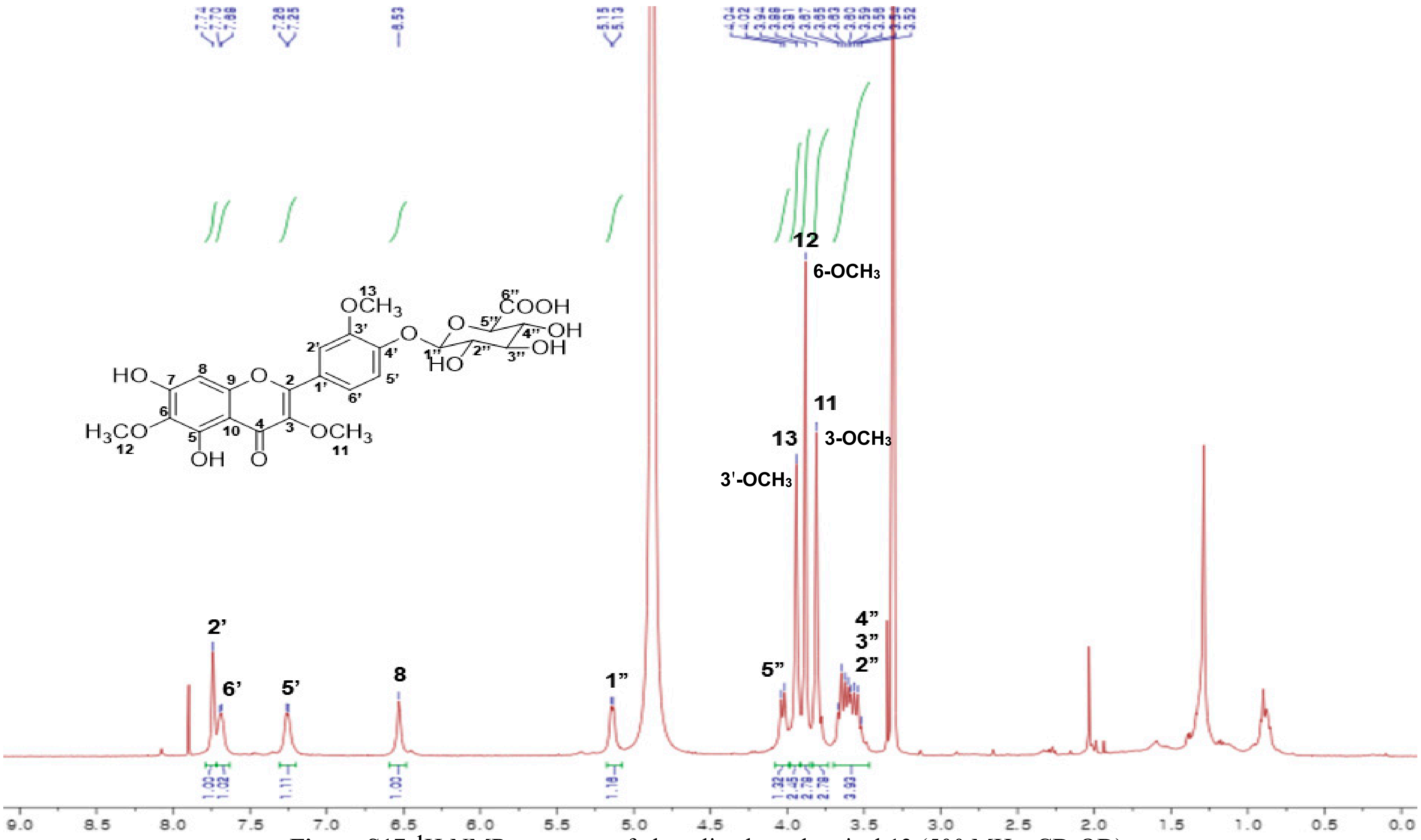

Figure S17. ^{1}H-NMR spectrum of phenolic phytochemical **12** (500 MHz, CD_3OD).

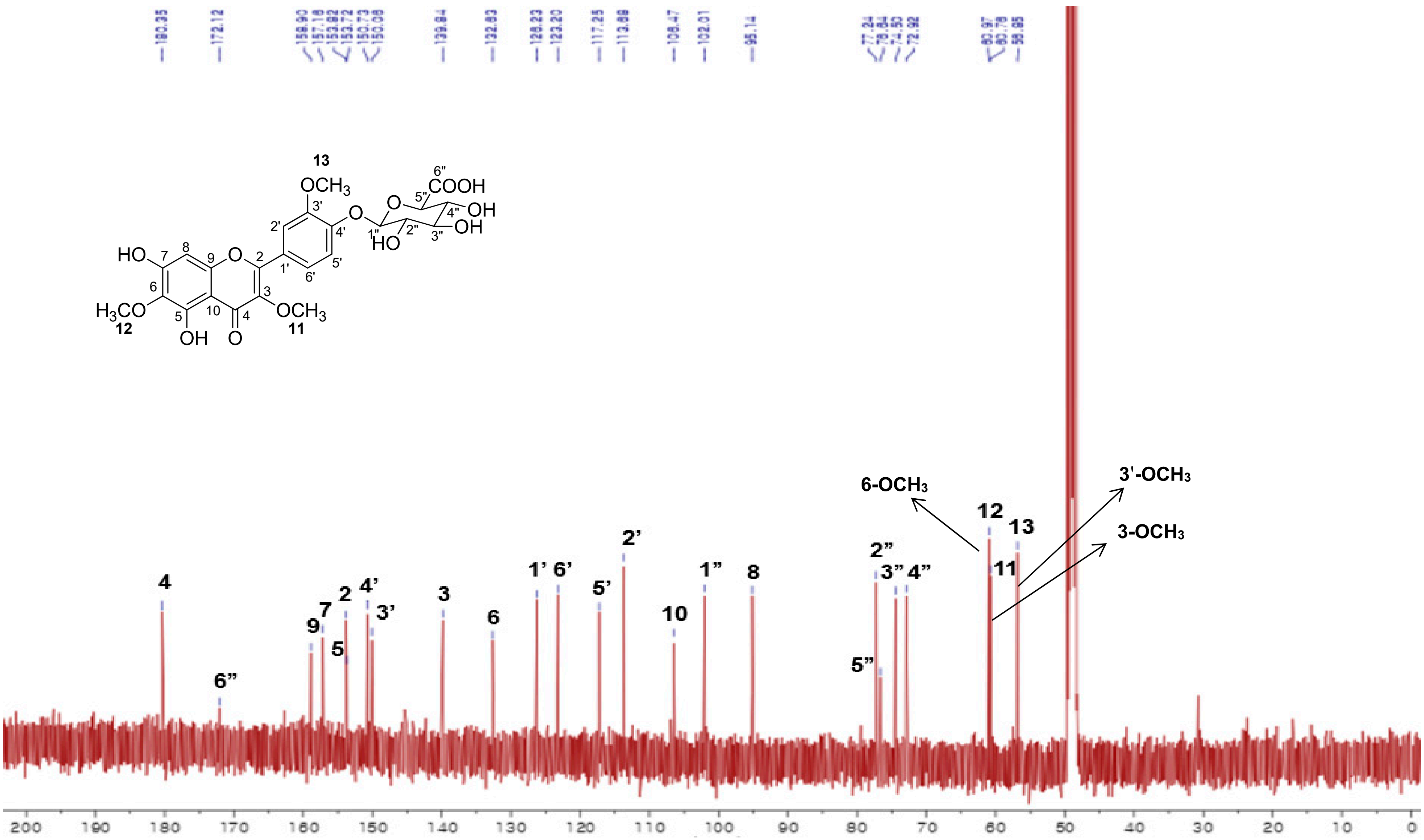

Figure S18. ^{13}C –NMR spectrum of phenolic phytochemical **12** (125 MHz, CD_3OD).

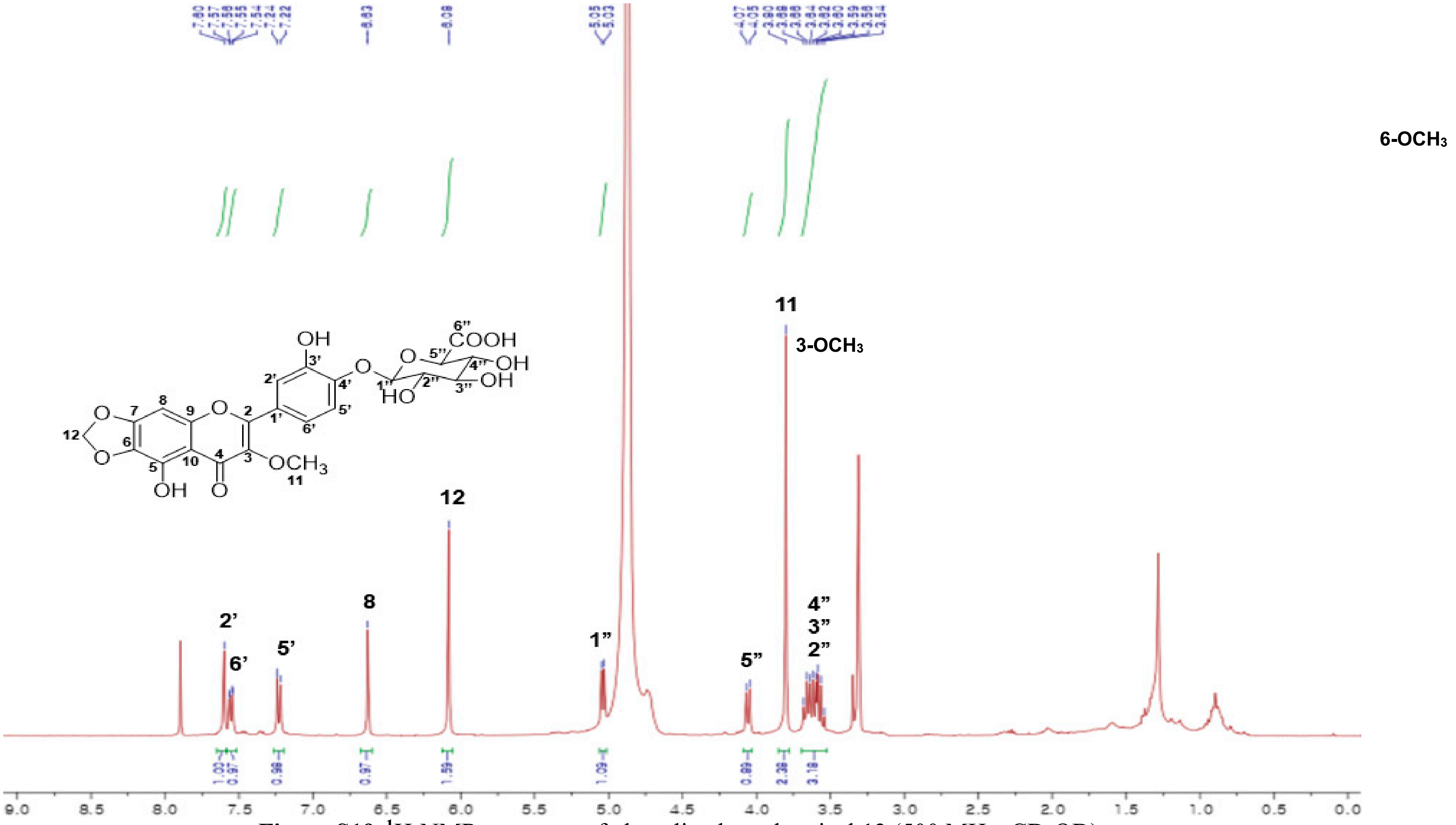

Figure S19. ^{1}H-NMR spectrum of phenolic phytochemical **13** (500 MHz, CD_3OD).

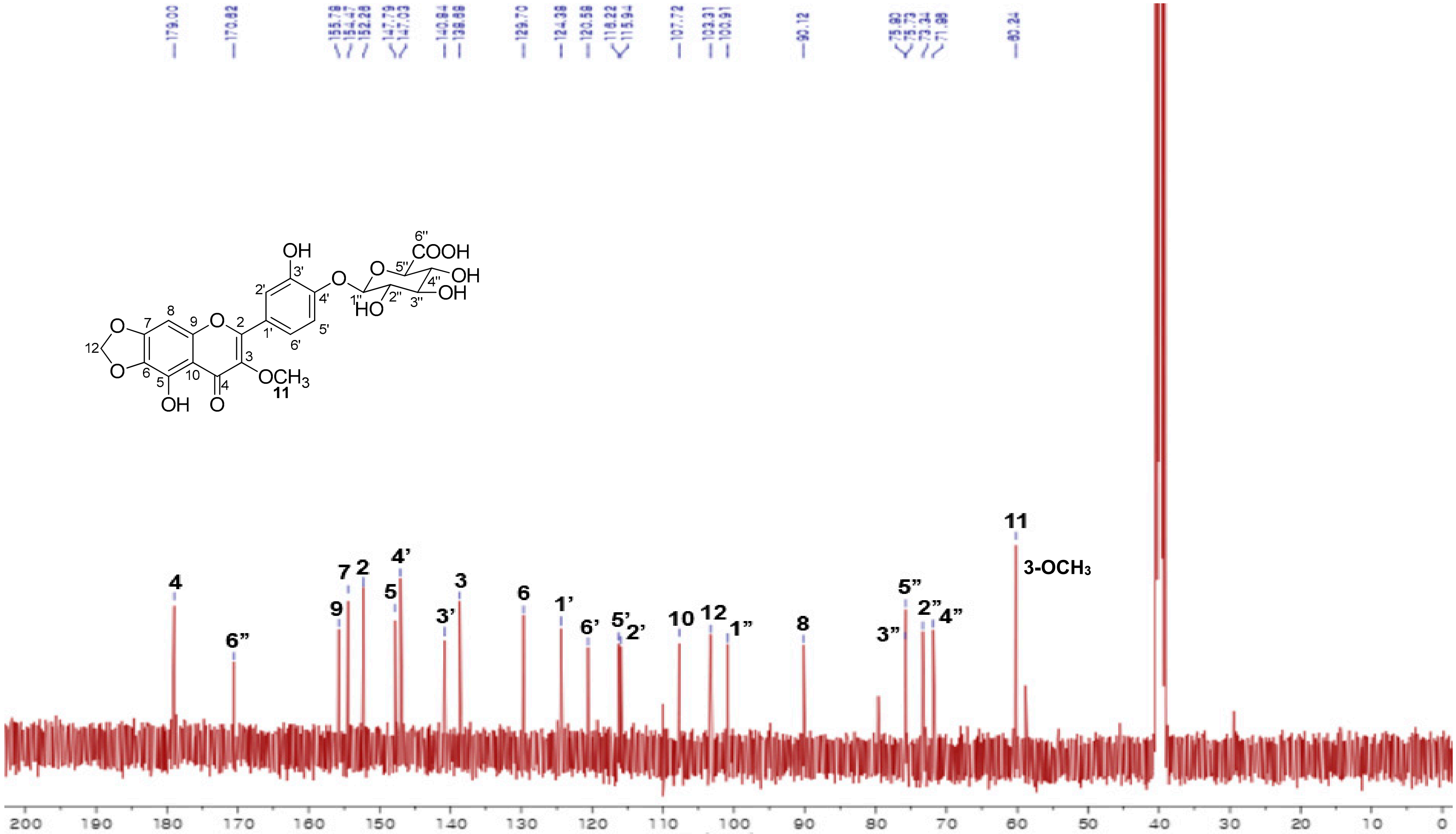

Figure S20. ^{13}C –NMR spectrum of phenolic phytochemical **13** (125 MHz, CD_3OD).
